# Testing the triple network model of psychopathology in a transdiagnostic neurodevelopmental cohort

**DOI:** 10.1016/j.nicl.2023.103539

**Published:** 2023-11-09

**Authors:** Jonathan S. Jones, Alicja Monaghan, Amelia Leyland-Craggs, the CALM Team, Duncan E. Astle

**Affiliations:** a MRC Cognition and Brain Sciences Unit, University of Cambridge, UK; b Department of Psychiatry, University of Cambridge, UK

**Keywords:** Neurodiversity, Hyperactivity, Inattention, ADHD, Functional Connectivity, Resting State Networks

## Abstract

**Aim:**

The triple network model of psychopathology posits that altered connectivity between the Salience (SN), Central Executive (CEN), and Default Mode Networks (DMN) may underlie neurodevelopmental conditions. However, this has yet to be tested in a transdiagnostic sample of young people.

**Method:**

We investigated this in 175 children (60 girls) that represent a heterogeneous population who are experiencing neurodevelopmental difficulties in cognition and behavior, and 60 comparison children (33 girls). Hyperactivity/impulsivity and inattention were assessed by parent-report. Resting-state functional Magnetic Resonance Imaging data were acquired and functional connectivity was calculated between independent network components and regions of interest. We then examined whether connectivity between the SN, CEN and DMN was dimensionally related to hyperactivity/impulsivity and inattention, whilst controlling for age, gender, and motion.

**Results:**

Hyperactivity/impulsivity was associated with increased functional connectivity between the SN, CEN, and DMN in at-risk children, whereas it was associated with decreased functional connectivity between the CEN and DMN in comparison children. These effects replicated in an adult parcellation of brain function and when using increasingly stringent exclusion criteria for in-scanner motion.

**Conclusion:**

Triple network connectivity characterizes transdiagnostic neurodevelopmental difficulties with hyperactivity/impulsivity. We suggest that this may arise from delayed network segregation, difficulties sustaining CEN activity to regulate behavior, and/or a heightened developmental mismatch between neural systems implicated in cognitive control relative to those implicated in reward/affect processing.

## Highlights

• Neurodevelopmental difficulties are linked to connectivity in key brain networks.
• Hyperactivity correlated with reduced network segregation in referred children.
• This differed in non-referred children, suggesting different adaptive mechanisms.

## 1 Introduction

Neurodevelopmental conditions affect up to 10% of children (NICE, 2019) but many more require additional support at school (Department for Education, 2021). These conditions vary widely in scope and impact, often being characterized in terms of cognition, behavior, communication, mental health, academic attainment, and lived experience. In recent years there has been a gradual shift away from seeking single common causes of individual disorder categories, towards the identification of mechanisms that may explain neurodevelopmental characteristics which span diagnostic boundaries (Astle and Fletcher-Watson, 2020, Astle et al., 2021, Kushki et al., 2019, Insel et al., 2010). One prominent example is the ‘triple network model of psychopathology’ (hereafter termed the ‘triple network model’), which postulates that atypical functional interactions between the Salience (SN), Central Executive (CEN) and Default-Mode Networks (DMN) underlie a range of neurodevelopmental, mental health, psychiatric, and neurodegenerative characteristics (Menon, 2011). This transdiagnostic model suggests that some common difficulties in mood and cognition could reflect alterations in the coupling between these networks. Support for this theory has largely come from independent diagnostically-based case-control studies, showing group differences in network interactions or correlations between these interactions and symptom severity. However, the model has yet to be tested in a transdiagnostic sample of children. This is an important next step because, if correct, the model would predict that neurodevelopmental difficulties reflect differential interactions between these three networks. Here, we test the triple network model in a broad heterogeneous sample of children with a range of diagnostic statuses who were referred for cognitive and behavioral difficulties. Specifically we tested whether functional connectivity between the SN, CEN and DMN is significantly associated with inattention and hyperactivity/impulsivity.

Interactions between the SN, CEN and DMN may help to characterize neurodevelopmental diversity because of their critical roles in higher-order cognition and goal-directed behavior (Menon, 2011). They are emergent properties of adult brain function (Yeo et al., 2011, Power et al., 2011, Power et al., 2012), develop in childhood (Chahal et al., 2022), and are linked to deviations in cognitive development (Sherman et al., 2014). The CEN is a lateral fronto-parietal network primarily consisting of the lateral prefrontal (lPFC) and posterior parietal cortices, and is broadly involved in cognitive control (Marek and Dosenbach, 2018 Jun). The DMN primarily comprises of the medial prefrontal cortex (mPFC), posterior cingulate (PC), precuneus and angular gyrus, and it is involved in emotion processing, self-referential thought, social cognition, and episodic memory (Raichle, 2015). Finally, the SN primarily comprises of the dorsal anterior cingulate (AC) and anterior insula (AI), and is involved in the detection of salient internal and external stimuli to adaptively guide behavior (Menon and Toga, 2015). The CEN is activated during externally-oriented cognitively demanding tasks whereas the DMN is typically deactivated. The SN has been shown to mediate this switch between cognition of external and internal stimuli (Sridharan et al., 2008).

To date, the triple network model has only been examined in case-control studies of Attention Deficit Hyperactivity Disorder (ADHD) and autism. These studies have reported functional connectivity differences within and between the SN, CEN, and DMN, which scale with symptom severity (Tomasi and Volkow, 2012, Sripada et al., 2014, Lin et al., 2021, Cai et al., 2018, Abbott et al., 2016, Nomi and Uddin, 2015). However, this altered functional connectivity may reflect a broader phenotype of behavioral characteristics that commonly occur in both ADHD and autism, rather than a diagnostic status per se. For example, hyperactivity/impulsivity and inattention are common across neurodevelopmental conditions (Arnett et al., 2018 Mar, van Steijn et al., 2012, Germanò et al., 2010, McClain et al., 2017), occurring in as many as 76% of autistic children (Joshi et al., 2014 Aug) and those without any formal diagnosis (Jones et al.., 2021). Transdiagnostic studies, with more inclusive sampling frameworks, therefore allow researchers to associate particular aspects of neurobiology with behavioral characteristics.

In the current study, we used a large transdiagnostic sample of children who experience difficulties in cognition and behavior, with variable scope and impact (Jones et al., 2021, Siugzdaite et al., 2020, Astle et al., 2019, Bathelt et al., 2018, Holmes et al., 2019, Holmes et al., 2020, Mareva and Holmes, 2019, Jones et al., 2021). Children were referred to the sample by professionals in children’s educational or clinical services, and are at elevated risk of educational underachievement (Gathercole et al., 2016), underemployment (Emerson and Hatton, 2008), and mental health difficulties (Emerson and Hatton, 2007). Hereafter, we refer to these children as neurodevelopmentally ‘at risk’. Importantly, children did not *need* a formal diagnosis to be referred to the sample, or conversely they could have *multiple* diagnoses. The crucial shared characteristic of the ‘at risk’ sample is they were defined functionally within educational or clinical services of experiencing difficulties in attention, learning, language or memory. We also included a comparison sample of children not referred on the basis of neurodevelopmental difficulties. We then tested whether functional connectivity between the SN, CEN and DMN is associated with parent-reported hyperactivity/impulsivity and inattention in each sample.

## 2 Materials and methods

### 2.1 Sample characteristics

Behavioral and resting-state functional Magnetic Resonance Imaging (fMRI) data were available for 343 children and adolescents who opted to take part in the MRI portion of the Centre for Attention, Learning and Memory (CALM) project (Holmes et al., 2019). This secondary analysis study was approved by the CALM Management Committee. Neurodevelopmentally at-risk children were identified by education and clinical practitioners as struggling in the areas of attention, learning, language, and memory. Comparison children were recruited from the same schools without such difficulties. Children with high motion scans (*n =* 108) were excluded from the analysis (see ‘fMRI Preprocessing’ for details). The final sample consisted of 235 children and adolescents aged 5–17 years: 175 at-risk and 60 comparison children (see Table 1). Important to note, just as a diagnosis is not an *inclusion* criteria for the ‘at risk’ sample, so too diagnosis is not an *exclusion* criteria for the comparison group. For instance, having received a diagnosis of dyslexia at some point does not mean that the child is currently struggling. Nonetheless, as readers might expect, despite not being inclusion or exclusion criteria, 66 children in the final ‘at risk’ sample, versus only three in the final comparison sample, have received a formal diagnosis of some kind.

**Table 1:** Group characteristics.

	All (*n =* 235)	At-risk (*n* = 175)	Comparison (*n =* 60)
Age in years: *M* (*SD*)	10.79 (2.17)	10.72 (2.20)	11.02 (2.08)
Boys: *n*	142 (60.42%)	115 (65.71%)	27 (45%)
Girls: *n*	93 (39.57%)	60 (34.29%)	33 (55%)
Ethnicity: *n*			
Asian/Asian British	0 (0%)	0 (0%)	0 (0%)
Black/African/Caribbean/Black British	1 (0.88%)	0 (0%)	1 (2.7%)
Mixed/Multiple Ethnic Groups	10 (8.85%)	8 (10.53%)	2 (5.41%)
White	102 (90.27%)	68 (89.47%)	34 (91.89%)
Any diagnosis^a^: *n*	69 (29.36%)	66 (37.71%)	3 (5%)
ADHD: *n*	33 (14.04%)	32 (18.29%)	1 (1.67%)
Autism: *n*	13 (5.53%)	13 (7.42%)	0 (0%)
Dyslexia: *n*	19 (8.09%)	17 (9.71%)	2 (3.33%)
IMD: *M* (*SD*)	21,505 (7771)	20,421 (7713)	24,556 (7152)
Hyperactivity/Impulsivity: *M* (*SD*)	8.12 (5.83)	9.65 (5.59)	3.65 (3.89)
Inattention: *M* (*SD*)	9.49 (5.08)	11.47 (3.68)	3.7 (4.08)
SN-CEN FC: *M* (*SD*)	0.10 (0.18)	0.11 (0.18)	0.05 (0.17)
SN-DMN FC: *M* (*SD*)	0.16 (0.23)	0.15 (0.23)	0.17 (0.22)
CEN-DMN FC: *M* (*SD*)	0.17 (0.18)	0.18 (0.18)	0.16 (0.17)
In-scanner motion: *M* (*SD*)	0.20 (0.09)	0.21 (0.09)	0.16 (0.08)

*Note*. Age at brain scan. The Index of Multiple Deprivation (IMD) was available for 229 children. Ethnicity data were available for 113 children. Attention Deficit Hyperactivity Disorder (ADHD), Salience Network (SN), Central Executive Network (CEN), Default Mode Network (DMN), and Functional Connectivity (FC).

a Any neurodevelopmental or mental health diagnosis.

## 3 Measures

### 3.1 Hyperactivity/impulsivity and inattention

The Conners Parent Rating Short Form 3rd Edition is a validated and reliable parent questionnaire of behavior in childhood that is widely used in clinical contexts (Conners, 2013). Parents or carers rated the frequency of 11 behavioral items over the past month that corresponded to the two scales of hyperactivity/impulsivity and inattention. The raw total score of each scale was used in subsequent analyses.

### 3.2 Image Acquisition

Magnetic resonance imaging data were acquired at the MRC Cognition and Brain Sciences Unit, University of Cambridge. All scans were obtained on a Siemens 3T Prisma-fit system (Siemens Healthcare, Erlangen, Germany), using a 32-channel quadrature head coil.

In the resting-state fMRI, 270 T2*-weighted whole-brain echo planar images (EPIs) were acquired over nine minutes (time repetition [TR] = 2 s; time echo [TE] = 30 ms; flip angle = 78 degrees, 3 × 3 × 3 mm). The first 4 volumes were discarded to ensure steady state magnetization. Participants were instructed to lie still with their eyes closed and to not fall asleep. For registration of functional images, T1-weighted volume scans were acquired using a whole-brain coverage 3D Magnetization Prepared Rapid Acquisition Gradient Echo (MP-RAGE) sequence acquired using 1-mm isometric image resolution (TR = 2.25 s, TE = 2.98 ms, flip angle = 9 degrees, 1x1x1mm).

### 3.3 fMRI pre-processing

The data were minimally pre-processed in fMRIPrep version 1.5.0 (Esteban et al., 2019), which implements slice-timing correction, rigid-body realignment, boundary-based co-registration to the structural T1, segmentation, and normalization to the MNI template. The data were then smoothed by 6 mm full-width at half-maximum. Strategies to denoise motion and physiological artefacts were evaluated in fmridenoise (Finc et al., 2019). The most effective strategy included regression of 24 head motion parameters (six rigid body realignment parameters, their squares, their derivatives, and their squared derivatives), 10 aCompCor components derived from the WM and CSF signal (Behzadi et al., 2007), linear and quadratic trends, motion spikes (framewise displacement > 0.5 mm; (Power et al., 2011), and a band-pass filter between 0.01 and 0.1 Hz (see (Jones et al.., 2021), for details). Simultaneous confound regression was performed in the Nipype (version 1.2.0) implementation of AFNI’s 3dTproject (Cox, 1996). Children who moved substantially during the scan were excluded from the analysis: first on the basis of high average motion (mean framewise displacement > 0.5 mm, *n* = 89) and then on the number of motion spikes (>20% spikes, *n* = 19), where few temporal degrees of freedom would have remained. The final sample included 235 children. Average in-scanner motion was 0.2 mm (*SD* = 0.09 mm).

### 3.4 Resting-state networks

**Fig. 1:**
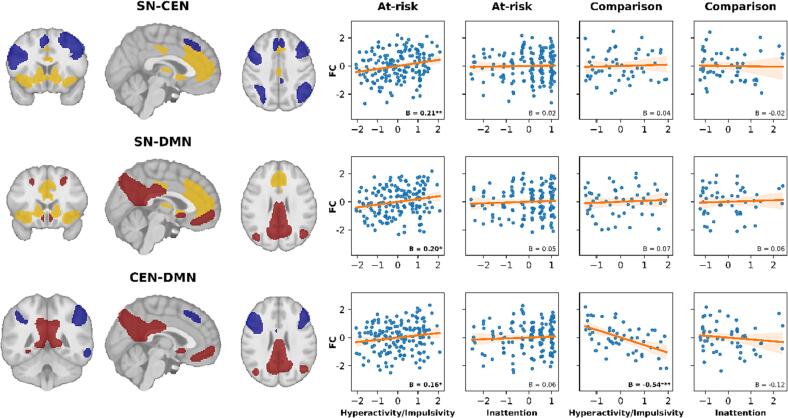
Associations between triple network connectivity and neurodevelopmental difficulties. Network masks derived from the ICA (left) are shown for the SN (yellow), CEN (blue), and DMN (red). Within-group partial regression plots (right) display the dimensional relations between behavioral difficulties and functional connectivity after age, age^2^, gender, and in-scanner motion have been regressed out. The shaded area around the regression line shows 95% confidence intervals from 1000 bootstrapped resamples. Salience Network (SN), Central Executive Network (CEN), and Default Mode Network (DMN). ***p < 0.001, **p < 0.01, *p < 0.05. (For interpretation of the references to colour in this figure legend, the reader is referred to the web version of this article.)

Spatial components of the denoised resting-state data were identified using canonical Independent Components Analysis on the whole sample using nilearn 0.7.0, with the number of components set to 30 (Varoquaux et al., 2010). We visually identified five components that corresponded with the SN, CEN, and DMN. The SN component included: the bilateral AC, medial orbitofrontal cortex (mOFC), bilateral ventral AI, caudate head, nucleus accumbens, and globus pallidus. The left CEN component included: the left lPFC, left frontal pole, left medial superior frontal gyrus, and left intraparietal sulcus (IPS). The right CEN component included: the right lPFC, right frontal pole, right medial superior frontal gyrus, and right IPS/inferior parietal lobe. The DMN component included: the mPFC, retrosplenial cortex, bilateral angular gyrus, bilateral superior frontal cortex and hippocampus. Finally, the posterior DMN component included: the bilateral PC/precuneus and bilateral IPS. Binary masks were created for each component and the DMN masks were combined (see Fig. 1). Time series were extracted from the four network masks and correlated using Pearson correlations to give a measure of functional connectivity between each pair of networks. Functional connectivity of the CEN with the SN and DMN was estimated as the average of the right and left CEN, respectively.

### 3.5 Adult networks and regions of interest

We also investigated functional connectivity between 14 key canonical regions of the SN, CEN, and DMN derived from a 100 region 17 networks parcellation of adult resting-brain function (Schaefer et al., 2018). This included bilateral regions of the SN (dorsal AI and dorsal AC), CEN (lPFC and IPS), and DMN (mPFC and PC/precuneus). In our analyses of the SN, we also included a region of the ventral AI; although this was assigned to the adult DMN, it aligns with both the data-driven SN component and the original description of the SN (Seeley et al., 2007, Seeley, 2019). We analyzed inter-regional functional connectivity within every network and between every pair of networks. Within each set of analyses, we corrected for multiple comparisons using the False Discovery Rate (FDR) Benjamini-Hochberg procedure (Benjamini and Hochberg, 1995). Finally, we analyzed functional connectivity between the adult networks by combining the regions into four network masks and correlating the extracted time-series: the dorsal SN (dorsal AI and dorsal AC), ventral SN (ventral AI and dorsal AC), CEN, and DMN.

### 3.6 Statistical analyses

Dimensional relationships between connectivity and behavior were investigated within each sample using ordinary least squares regression whilst controlling for age, age^2^, gender, and in-scanner motion. Put simply, we wanted to test whether any of the inter-network connectivity metrics were significantly associated with continuous measures of behavior, both in the referred ‘at risk’ sample, and in the non-referred comparison sample. We contrasted the dimensional effects across the two samples by including sample-by-behavior dimension as an interaction term in the model. Next, we did a more conventional categorical group comparison, again using ordinary least squares regression because it allowed us to control for the various covariates. We made two categorical contrasts, first contrasting everyone in the at-risk sample with everyone in the comparison sample, second more specifically contrasting everyone with a combined type ADHD diagnosis, relative to the comparison sample. Important to note: these two categorical contrasts are necessarily overlapping, because the at-risk sample will include the children with the ADHD diagnoses. Univariate statistical outliers greater than or less than three times the median absolute deviation were excluded from each analysis in a casewise manner, as in previous studies (Cai et al., 2018). All analysis code is available at: https://osf.io/7mptz/ (https://doi.org/10.17605/OSF.IO/7MPTZ).

## 4 Results

### 4.1 Triple network connectivity and dimensions of behaviour

Within the neurodevelopmentally at-risk sample, hyperactivity/impulsivity was significantly associated with greater functional connectivity between the SN-CEN (n = 172, *β* = 0.21, p = 0.006), SN-DMN (n = 164, *β* = 0.20, p = 0.013), and CEN-DMN (n = 166, *β* = 0.16, p = 0.042). However, these associations were not apparent in the comparison sample (see Fig. 1 and Tables S1-S2); instead, hyperactivity/impulsivity was significantly associated with reduced functional connectivity between the CEN-DMN (n = 53, *β =* −0.54, p = 6.7E-05), and this brain-behavior association significantly differed between the groups (n = 219, *β* = 0.65, p = 4.89E-05).

Within the at-risk sample we further tested whether these relationships between functional connectivity and hyperactivity were particularly driven by those with an ADHD diagnosis. We did this by contrasting the slopes (Cohen et al., 2003). However, there was no significant difference between these slopes for those with versus without a diagnosis of ADHD for the SN-CEN (t(171) = 1.302, p = 0.097), SN-DMN (t(171) = 0.109, p = 0.457) or CEN-DMN (t(171) = 0.568, p = 0.285). We also tested whether the relationships between functional connectivity and hyperactivity were particularly driven by those with *any* formal diagnosis. There was no significant difference between the slopes of those *with* versus *without* any diagnosis for the SN-CEN (t(171) = 0.387, p = 0.350) or SN-DMN (t(171) = 1.463, p = 0.073), but there was for the CEN-DMN (t(171) = 1.814, p = 0.036). The latter stemmed from a steeper slope in those with a diagnosis (n = 66, *β* = 0.371, p = 0.009), versus those without (n = 105, *β* = 0.083, p = 0.392). The full results of this sensitivity analysis can be found in Table S3.

We then evaluated whether these effects generalized to a common parcellation of adult resting brain function (see Tables S4-S5). In the at-risk sample, hyperactivity/impulsivity was again significantly associated with greater CEN-DMN connectivity (*n =* 169, *β =* 0.21, *p =* 0.009) and greater SN-DMN connectivity (ventral SN: *n =* 171, *β =* 0.2, *p =* 0.009; dorsal SN: *n =* 166, *β =* 0.15, *p =* 0.05), but not SN-CEN connectivity (ventral SN: *n =* 160, *β =* 0.03, *p =* 0.723; dorsal SN: *n =* 164, *β =* −0.07, *p =* 0.373). As before, there was a significant group interaction on the association between hyperactivity/impulsivity and CEN-DMN connectivity (*n =* 222, *β =* 0.37, *p =* 0.021).

### 4.2 Group differences

Group differences also implicated elevated SN-CEN functional connectivity with neurodevelopmental difficulties (see Tables S6-S7). Specifically, SN-CEN connectivity was greater in children diagnosed with combined type ADHD (*n =* 32, *M* = 0.16, *SD* = 0.13) than comparison children (*n =* 58, *M* = 0.06, *SD* = 0.16); *β =* 0.56, *p =* 0.023. SN-CEN connectivity was also greater in the neurodevelopmentally at-risk sample as a whole (*n =* 172, *M* = 0.11, *SD* = 0.17) than comparison children (*n =* 59, *M* = 0.06, *SD* = 0.16); however, this was not significant (*β =* 0.19, *p =* 0.220).

### 4.3 Regional connectivity

**Fig. 2:**
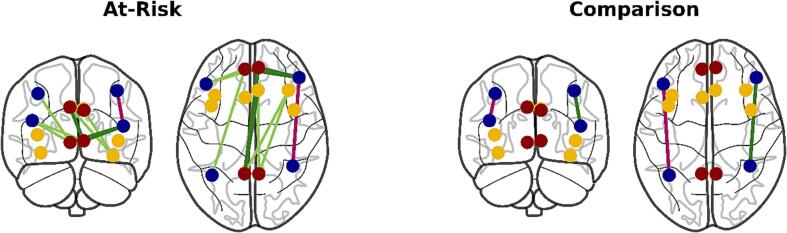
Associations between hyperactivity/impulsivity and inter-regional functional connectivity in the Salience (yellow), Central Executive (blue), and Default Mode Networks (red). Green lines indicate positive associations and pink lines indicate negative associations. Borderline significant associations are shown in lighter colors with less weight (*p <* 0.073). (For interpretation of the references to colour in this figure legend, the reader is referred to the web version of this article.)

In line with the network level effects, increasing functional connectivity between core regions of the SN, CEN, and DMN was associated with more marked hyperactivity/impulsivity in the at-risk sample, but not the comparison sample (see Fig. 2 and Tables S8-13). Between the CEN and DMN, hyperactivity/impulsivity was significantly associated with greater connectivity between the right lPFC and right mPFC (*n =* 172, *β =* 0.25, *p =* 0.008 FDR-corrected) and the right lPFC and left mPFC (*n =* 172, *β =* 0.29, *p =* 0.003 FDR-corrected). Between the SN and DMN, hyperactivity/impulsivity was associated with greater connectivity between the right dorsal AC and left PC (*n =* 170, *β =* 0.26, *p =* 0.028 FDR-corrected).

Within networks, reduced functional connectivity within the CEN and increased connectivity within the DMN was associated with more marked hyperactivity/impulsivity in the at-risk sample (see Tables S14-19). In the CEN, hyperactivity/impulsivity was associated with decreased connectivity between the right lPFC and right IPS (*n =* 162, *β =* −0.22, *p =* 0.04 FDR-corrected). In the DMN, hyperactivity/impulsivity was associated with increased connectivity between the right mPFC and left PC (*n =* 163, *β =* 0.22, *p =* 0.042 FDR-corrected). In the comparison sample, the associations between regional CEN functional connectivity and hyperactivity/impulsivity were mixed. Consistent with the at-risk sample, greater connectivity between the *left* lPFC and *left* IPS was associated with *less* hyperactivity/impulsivity (*n =* 53, *β =* −0.35, *p =* 0.046 FDR-corrected). But in direct contrast with the at-risk sample, and as indicated by significant group interaction (*n =* 216, *β =* 0.44, *p =* 0.045 FDR-corrected), greater connectivity between the *right* lPFC and *right* IPS was associated with *greater* hyperactivity/impulsivity in the comparison sample (*n =* 54, *β =* 0.36, *p =* 0.046 FDR-corrected).

### 4.4 Robustness analyses

As our findings pertained to hyperactivity, we opted to further rule out the confounding effects of in-scanner motion by repeating our main analyses after excluding children with mean framewise displacement above 0.15–0.45 mm. Overall, effect sizes were similar and, despite limited power in these smaller samples, we were able to replicate our findings at multiple thresholds (see Tables S20-27).

## 5 Discussion

In the current study we tested whether the triple network model, which implicates divergent connectivity between the SN, CEN, and DMN in a range of cognitive and affective conditions, can characterize behavioral difficulties in a broad transdiagnostic sample of children. Hyperactivity/impulsivity across this sample of neurodevelopmentally at-risk children was associated with increased connectivity between the SN, CEN, and DMN. We replicated these effects using networks and regions derived from an adult parcellation of brain function. These findings provide direct transdiagnostic evidence for the triple network model in a neurodevelopmental sample. Interestingly, these associations differed in a sample of comparison children, particularly between the CEN-DMN and within the CEN, suggesting different neurodevelopmental origins. Finally, we replicated our findings when excluding children who moved in the scanner at increasingly stringent thresholds.

### 5.1 Transdiagnostic evidence for the triple network model in neurodevelopmental risk

We found novel evidence for the triple network model (Menon, 2011) in a mixed sample of children with diverse neurodevelopmental characteristics and diagnoses. Importantly, we showed that hyperactivity/impulsivity was associated with higher functional connectivity between all three networks in neurodevelopmentally at-risk children. Larger deviations in functional connectivity were related to a greater degree of neurodevelopmental difficulties. The relationship between CEN-DMN connectivity and hyperactivity/impulsivity was particularly strong in those with *any* diagnosis, but other than that, the pattern of effects was not significantly differentiated by diagnosis. In contrast, previous case-control studies have implied that deviations in connectivity are related to the specific symptoms of ADHD or autism (Cai et al., 2018, Abbott et al., 2016). Our findings were robust to different parcellation methods; we demonstrated that hyperactivity/impulsivity was also associated with greater CEN-DMN and SN-DMN connectivity in the at-risk sample when using regions and networks derived from an adult atlas of functional connectivity. Interestingly, we found less evidence for categorical group differences in connectivity: only SN-CEN functional connectivity was significantly elevated in those diagnosed with combined type ADHD. One possible reason for this may be the substantial heterogeneity in the types and degree of neurodevelopmental difficulties in our at-risk sample (Jones et al., 2021) and children with ADHD (Astle et al., 2021), which was better captured by the dimensional analyses. In short, hyperactivity/impulsivity was related to elevated connectivity between the SN, CEN and DMN. But, why might this elevated connectivity occur in neurodevelopment?

### 5.2 Functional segregation may underlie neurodevelopmental difficulties

The developmental process of functional segregation (decreased FC) may diverge in children experiencing neurodevelopmental difficulties. In childhood, brain function increasingly segregates into local densely connected neighborhoods. The connections between segregated communities are not diffuse, but they are instead integrated at the global level through networks of coordinated regions (Fair et al., 2013, Satterthwaite et al., 2013). This phenomenon leads to *decreasing* overall functional connectivity *between* the SN, CEN and DMN through childhood and adolescence (Chahal et al., 2022, Sherman et al., 2014, Chai et al., 2014, Ellwood-Lowe et al., 2021, Jolles et al., 2020). The elevated connectivity in hyperactive/impulsive individuals would therefore be similar to younger individuals without these difficulties. Indeed, previous evidence in this at-risk cohort demonstrated that the CEN and a limbic network, which overlaps with the SN component in the mOFC, did not segregate with age relative to comparison children (Gathercole et al., 2016). Where the development of these networks is attenuated or delayed, it may act as a particular risk factor for hyperactivity/impulsivity, consistent with evidence in children with a formal ADHD diagnosis (Sripada et al., 2014, Kessler et al., 2016).

Reduced CEN-DMN segregation and reduced CEN integration (that is, reduced positive FC between regions of the CEN) in children with the most marked hyperactivity/impulsivity may stem from a history of difficulties sustaining CEN activity. The CEN-DMN finding was evidenced in the data-driven networks, adult networks, and between specific regions. It is consistent with many previous studies implicating higher CEN-DMN connectivity in neurodevelopmental difficulties, including hyperactivity/impulsivity and inattention (Lin et al., 2018, Barber et al., 2015, Gao et al., 2019), IQ (Sherman et al., 2014, Ellwood-Lowe et al., 2021), and inhibitory control (Barber et al., 2013). The CEN regulates cognitive control by coordinating externally oriented networks and suppressing the internally oriented default-mode network (Marek and Dosenbach, 2018 Jun). Higher resting CEN-DMN functional connectivity has been implicated in lapses of attention and may reflect a history of difficulties maintaining CEN activity and suppressing DMN activity during attention demanding tasks (Sonuga-Barke and Castellanos, 2007). Extending this theory, difficulties sustaining CEN activity may also result in failures to inhibit inappropriate behaviors (Zhang et al., 2017), leading to impulsive actions and increased motor activity. Although we found no significant association with inattention, others have (Cai et al., 2018, Gao et al., 2019) and we suspect that this is due to a ceiling effect on this specific measure in our sample.

Relatedly, a greater propensity for impulsive behavior in at-risk children could originate from a heightened developmental mismatch between the SN and CEN. The developmental mismatch hypothesis explains how increased sensation seeking and risk-taking behavior in adolescence may arise from the protracted development of cognitive control in the CEN, relative to the early development of affect and reward processing in limbic and subcortical regions, such as the medial OFC and nucleus accumbens (Casey et al., 2008, Somerville et al., 2010, Steinberg, 2008, Shulman et al., 2016). Notably, these regions were only included in the data-driven SN component, where SN-CEN connectivity was associated with hyperactivity/impulsivity. Greater connectivity between the SN and CEN components is consistent with the idea that regions involved in emotion and reward processing are under-regulated by the CEN, which is in turn linked to impulsive behavior (Zhai et al., 2015). Therefore, children with the greatest neurodevelopmental difficulties could have a heightened developmental mismatch.

### 5.3 Diverging brain-behavior associations

A consistent and surprising finding was that the at-risk and comparison samples had opposing associations between hyperactivity/impulsivity and CEN-DMN connectivity. As resting-state connectivity likely develops through interactive specialization that is dependent on co-activity between regions (Johnson, 2011), then network interactions can adaptively develop through different means and may underlie the distinct associations we observed in the two samples. This was demonstrated in a recent study where CEN-DMN connectivity was positively or negatively related to cognitive ability depending on whether the sample of children were socioeconomically at-risk or not (Ellwood-Lowe et al., 2021).

Better segregation between the CEN and DMN could be adaptive for children experiencing relatively high levels of hyperactivity and impulsivity. Indeed, it might afford some kind of regulatory process, perhaps using specialized regions to inhibit DMN activity in situations requiring externally oriented cognitive control (Barber et al., 2015). Conversely, for children with lower levels of hyperactivity and impulsivity this segregation may be less critical. Indeed, higher CEN-DMN connectivity could be cognitively advantageous for children who rely more on typical DMN functions, such as autobiographical memory and future planning, in cognitively demanding situations (Buckner and Carroll, 2007). Put simply, the role of network segregation could be somewhat different across these two samples.

It is important to note that even the highest hyperactivity/impulsivity score in comparison children was below the average hyperactivity/impulsivity score in at-risk children. Consequently, our finding may reflect that the true relationship between CEN-DMN connectivity and hyperactivity/impulsivity across the samples is non-linear. That is, when connectivity is very low or very high it is associated high levels of hyperactivity/impulsivity.

### 5.4 Limitations and future directions

We would like to highlight several limitations and future directions for our work. First, our findings pertained to only one measure of hyperactivity/impulsivity and future studies should address the specificity or generality to other neurodevelopmental difficulties. We suspect that this association was not specific and that the measure captured neurodevelopmental difficulties, such as behavioral regulation, more broadly. Second, we note the well-known limitations of self-report data but emphasize that these have clinical utility in the assessment of neurodevelopmental difficulties. Third, we extracted network components by performing a group ICA jointly across both samples. This practically enabled the direct comparison of brain coordinates across individuals, but it does not take into account spatial variation in functional networks within individuals or within samples.

## 6 Conclusion

We demonstrate that the triple network model differentiates behavioral difficulties across a transdiagnostic neurodevelopmental cohort. Specifically, we observed that functional segregation between these networks was generally attenuated in those with the greatest neurodevelopmental difficulties. We suggest multiple mechanisms that may contribute to this, including: delayed development of functional networks, a history of difficulties maintaining CEN activity, and a heightened developmental mismatch between neural systems responsible for cognitive control compared to those for reward/affect processing.

## 7 Disclosures

For the purpose of open access, the authors have applied a Creative Commons Attribution (CC-BY) license to any Author Accepted Manuscript version arising from this submission. A preprint of this article has been published at: 10.1101/2022.05.05.22274709.

## CRediT authorship contribution statement

**Jonathan S. Jones:** Conceptualization, Methodology, Software, Validation, Formal analysis, Investigation, Data curation, Writing – original draft, Writing – review & editing, Supervision, Visualization. **Alicja Monaghan:** Formal analysis, Visualization. **Amelia Leyland-Craggs:** Formal analysis. **the CALM Team:** Investigation, Resources, Data curation, Project administration, Funding acquisition. **Duncan E. Astle:** Conceptualization, Writing – original draft, Writing – review & editing, Supervision, Project administration, Funding acquisition.

## Declaration of Competing Interest

The authors declare the following financial interests/personal relationships which may be considered as potential competing interests: The authors report no potential conflicts of interest. The lead author (JJ) was subsequently employed by AstraZeneca as a Statistician following the completion of this work.

## Data Availability

The authors do not have permission to share data.

## References

[b0005] Abbott A.E., Nair A., Keown C.L., Datko M., Jahedi A., Fishman I. (2016). Patterns of atypical functional connectivity and behavioral links in autism differ between default, salience, and executive networks. Cereb. Cortex.

[b0010] Arnett A.B., Cairney B.E., Wallace A.S., Gerdts J., Turner T.N., Eichler E.E. (2018 Mar). Comorbid symptoms of inattention, autism, and executive cognition in youth with putative genetic risk. J. Child Psychol. Psychiatry.

[b0015] Astle D.E., Bathelt J., Holmes J. (2019). Remapping the cognitive and neural profiles of children who struggle at school. Dev. Sci..

[b0020] Astle D.E., Fletcher-Watson S. (2020). Beyond the core-deficit hypothesis in developmental disorders. Curr. Dir. Psychol. Sci..

[b0025] Astle D.E., Holmes J., Kievit R., Gathercole S.E. (2021). Annual Research Review: The transdiagnostic revolution in neurodevelopmental disorders. J Child Psychol. Psychiatry.

[b0030] Barber A.D., Caffo B.S., Pekar J.J., Mostofsky S.H. (2013). Developmental changes in within- and between-network connectivity between late childhood and adulthood. Neuropsychologia.

[b0035] Barber A.D., Jacobson L.A., Wexler J.L., Nebel M.B., Caffo B.S., Pekar J.J. (2015). Connectivity supporting attention in children with attention deficit hyperactivity disorder. Neuroimage Clin..

[b0040] Bathelt J., Holmes J., Astle D.E., Holmes J., Gathercole S., Astle D. (2018). Data-driven subtyping of executive function-related behavioral problems in children. J. Am. Acad. Child Adolesc. Psychiatry.

[b0045] Behzadi Y., Restom K., Liau J., Liu T.T. (2007). A component based noise correction method (CompCor) for BOLD and perfusion based fMRI. Neuroimage.

[b0050] Benjamini Y., Hochberg Y. (1995). Controlling the false discovery rate: a practical and powerful approach to multiple testing. J. R. Stat. Soc. Ser. B.

[b0055] Buckner R.L., Carroll D.C. (2007). Self-projection and the brain. Trends Cogn. Sci..

[b0060] Cai W., Chen T., Szegletes L., Supekar K., Menon V. (2018). Aberrant time-varying cross-network interactions in children with attention-deficit/hyperactivity disorder and the relation to attention deficits. Biol. Psychiatry Cogn. Neurosci. Neuroimaging.

[b0065] Casey B.J., Getz S., Galvan A. (2008). The adolescent brain. Dev. Rev..

[b0070] Chahal R., Miller J.G., Yuan J.P., Buthmann J.L., Gotlib I.H. (2022). An exploration of dimensions of early adversity and the development of functional brain network connectivity during adolescence: Implications for trajectories of internalizing symptoms. Dev. Psychopathol..

[b0075] Chai X.J., Ofen N., Gabrieli J.D.E., Whitfield-Gabrieli S. (2014). Selective development of anticorrelated networks in the intrinsic functional organization of the human brain. J. Cogn. Neurosci..

[b0080] Cohen J., Cohen P., West S.G., Aiken L.S. (2003).

[b0085] Conners C.K. (2013).

[b0090] Cox R.W. (1996). AFNI: Software for analysis and visualization of functional magnetic resonance neuroimages. Comput. Biomed. Res..

[b0095] Department for Education. Special educational needs and disability: an analysis and summary of data sources [Internet]. DfE London; 2021. Available from: https://assets.publishing.service.gov.uk/government/uploads/system/uploads/attachment_data/file/985162/Special_educational_needs_Publication_May21_final.pdf.

[b0100] Ellwood-Lowe M.E., Whitfield-Gabrieli S., Bunge S.A. (2021). Brain network coupling associated with cognitive performance varies as a function of a child’s environment in the ABCD study. Nat. Commun..

[b0105] Emerson, E., Hatton, C. 2008. CEDR Research Report 2008 (1): People with Learning Disabilities in England.

[b0110] Emerson E., Hatton C. (2007). Mental health of children and adolescents with intellectual disabilities in Britain. Br. J. Psychiatry.

[b0115] Esteban O., Markiewicz C.J., Blair R.W., Moodie C.A., Isik A.I., Erramuzpe A. (2019). fMRIPrep: a robust preprocessing pipeline for functional MRI. Nat. Methods.

[b0120] Fair D.A., Nigg J.T., Iyer S., Bathula D., Mills K.L., Dosenbach N.U.F. (2013). Distinct neural signatures detected for ADHD subtypes after controlling for micro-movements in resting state functional connectivity MRI data. Front. Syst. Neurosci..

[b0125] Finc K., Chojnowski M., Bonna K. (2019). fMRIDenoise: automated denoising, denoising strategies comparison, and functional connectivity data quality control. Zenodo.

[b0130] Gao Y., Shuai D., Bu X., Hu X., Tang S., Zhang L. (2019). Impairments of large-scale functional networks in attention-deficit/hyperactivity disorder: a meta-analysis of resting-state functional connectivity. Psychol. Med..

[b0135] Gathercole S.E., Woolgar F., Kievit R.A., Astle D., Manly T., Holmes J. (2016). How common are WM deficits in children with difficulties in reading and mathematics?. J. Appl. Res. Mem. Cogn. [Internet].

[b0140] Germanò E., Gagliano A., Curatolo P. (2010). Comorbidity of ADHD and dyslexia. Dev. Neuropsychol..

[b0145] Holmes J., Bryant A., Gathercole S.E. (2019). Protocol for a transdiagnostic study of children with problems of attention, learning and memory (CALM). BMC Pediatr..

[b0150] Holmes J., Guy J., Kievit R.A., Bryant A., Mareva S., Gathercole S.E. (2020). Cognitive dimensions of learning in children with problems in attention, learning, and memory. J. Educ. Psychol..

[b0155] Insel T., Cuthbert B., Garvey M., Heinssen R., Pine D.S., Quinn K. (2010). Research domain criteria (RDoC): toward a new classification framework for research on mental disorders. Am. J. Psychiatry.

[b0160] Johnson M.H. (2011). Interactive specialization: a domain-general framework for human functional brain development?. Dev. Cogn. Neurosci..

[b0165] Jolles D.D., Mennigen E., Gupta M.W., Hegarty C.E., Bearden C.E., Karlsgodt K.H. (2020). Relationships between intrinsic functional connectivity, cognitive control, and reading achievement across development. Neuroimage.

[b0170] Jones J.S., Astle D.E., The CALM Team (2021). A transdiagnostic data-driven study of children’s behaviour and the functional connectome. Dev. Cogn. Neurosci..

[b0175] Jones J.S., Astle D.E., The CALM Team (2021). Segregation and integration of the functional connectome in neurodevelopmentally “at risk” children. Dev. Sci..

[b0180] Joshi G., Faraone S.V., Wozniak J., Tarko L., Fried R., Galdo M. (2014). Symptom profile of ADHD in youth with high-functioning autism spectrum disorder: A comparative study in psychiatrically referred populations. J. Atten. Disord..

[b0185] Kessler D., Angstadt M., Sripada C.S. (2016). Growth charting of brain connectivity networks and the identification of attention impairment in youth. JAMA Psychiat..

[b0190] Kushki A., Anagnostou E., Hammill C., Duez P., Brian J., Iaboni A. (2019). Examining overlap and homogeneity in ASD, ADHD, and OCD: a data-driven, diagnosis-agnostic approach. Transl. Psychiatry [Internet]..

[b0195] Lin H.-Y., Kessler D., Tseng W.-Y.-I., Gau S.-S.-F. (2021). Increased functional segregation related to the salience network in unaffected siblings of youths with attention-deficit/hyperactivity disorder. J. Am. Acad. Child Adolesc. Psychiatry.

[b0200] Lin H., Lin Q., Li H., Wang M., Chen H., Liang Y. (2018). Functional connectivity of attention-related networks in drug-naïve children with ADHD. J. Atten. Disord..

[b0205] Marek S., Dosenbach N.U.F. (2018). The frontoparietal network: function, electrophysiology, and importance of individual precision mapping. Dialogues Clin. Neurosci..

[b0210] Mareva S., Holmes J. (2019). Transdiagnostic associations across communication, cognitive, and behavioural problems in a developmentally at-risk population: a network approach. BMC Pediatr..

[b0215] McClain M.B., Hasty Mills A.M., Murphy L.E. (2017). Inattention and hyperactivity/impulsivity among children with attention-deficit/hyperactivity-disorder, autism spectrum disorder, and intellectual disability. Res. Dev. Disabil..

[b0220] Menon V. (2011). Large-scale brain networks and psychopathology: A unifying triple network model. Trends Cogn. Sci. [Internet]..

[b0225] Menon V., Toga A.W. (2015). Brain Mapping: An Encyclopedic Reference (Vol 2).

[b0230] NICE. Attention deficit hyperactivity disorder: diagnosis and management [Internet]. 2019. Available from: https://www.nice.org.uk/guidance/ng87. 29634174

[b0235] Nomi J.S., Uddin L.Q. (2015). Developmental changes in large-scale network connectivity in autism. NeuroImage Clin..

[b0240] Power J.D., Cohen A.L., Nelson S.M., Wig G.S., Barnes K.A., Church J.A. (2011). Functional network organization of the human brain. Neuron.

[b0245] Power J.D., Barnes K.A., Snyder A.Z., Schlaggar B.L., Petersen S.E. (2012). Spurious but systematic correlations in functional connectivity MRI networks arise from subject motion. Neuroimage.

[b0250] Raichle M.E. (2015). The brain’s default mode network. Annu. Rev. Neurosci..

[b0255] Satterthwaite T.D., Wolf D.H., Ruparel K., Erus G., Elliott M.A., Eickhoff S.B. (2013). Heterogeneous impact of motion on fundamental patterns of developmental changes in functional connectivity during youth. Neuroimage.

[b0260] Schaefer A., Kong R., Gordon E.M., Laumann T.O., Zuo X.-N., Holmes A.J. (2018). Local-global parcellation of the human cerebral cortex from intrinsic functional connectivity MRI. Cereb. Cortex.

[b0265] Seeley W.W. (2019). The salience network: a neural system for perceiving and responding to homeostatic demands. J. Neurosci..

[b0270] Seeley W.W., Menon V., Schatzberg A.F., Keller J., Glover G.H., Kenna H. (2007). Dissociable intrinsic connectivity networks for salience processing and executive control. J. Neurosci..

[b0275] Sherman L.E., Rudie J.D., Pfeifer J.H., Masten C.L., McNealy K., Dapretto M. (2014). Development of the Default Mode and Central Executive Networks across early adolescence: A longitudinal study. Dev. Cogn. Neurosci..

[b0280] Shulman E.P., Smith A.R., Silva K., Icenogle G., Duell N., Chein J. (2016). The dual systems model: Review, reappraisal, and reaffirmation. Dev. Cogn. Neurosci..

[b0285] Siugzdaite R., Bathelt J., Holmes J., Astle D.E. (2020). Transdiagnostic brain mapping in developmental disorders. Curr. Biol..

[b0290] Somerville L.H., Jones R.M., Casey B.J. (2010). A time of change: Behavioral and neural correlates of adolescent sensitivity to appetitive and aversive environmental cues. Brain Cogn..

[b0295] Sonuga-Barke E.J., Castellanos F.X. (2007). Spontaneous attentional fluctuations in impaired states and pathological conditions: a neurobiological hypothesis. Neurosci. Biobehav. Rev..

[b0300] Sridharan D., Levitin D.J., Menon V. (2008). A critical role for the right fronto-insular cortex in switching between central-executive and default-mode networks. Proc. Natl. Acad. Sci. U.S.A..

[b0305] Sripada C.S., Kessler D., Fang Y., Welsh R.C., Prem Kumar K., Angstadt M. (2014). Disrupted network architecture of the resting brain in attention-deficit/hyperactivity disorder. Hum. Brain Mapp. [Internet].

[b0310] Sripada C.S., Kessler D., Angstadt M. (2014). Lag in maturation of the brain’s intrinsic functional architecture in attention-deficit/hyperactivity disorder. Proc. Natl. Acad. Sci. U. S. A..

[b0315] Steinberg L. (2008). A social neuroscience perspective on adolescent risk-taking. Dev. Rev..

[b0320] Tomasi D., Volkow N.D. (2012). Abnormal functional connectivity in children with attention-deficit/hyperactivity disorder. Biol. Psychiatry.

[b0325] van Steijn D.J., Richards J.S., Oerlemans A.M., de Ruiter S.W., van Aken M.A.G., Franke B. (2012). The co-occurrence of autism spectrum disorder and attention-deficit/hyperactivity disorder symptoms in parents of children with ASD or ASD with ADHD. J. Child Psychol. Psychiatry.

[b0330] Varoquaux G., Sadaghiani S., Pinel P., Kleinschmidt A., Poline J.B., Thirion B. (2010). A group model for stable multi-subject ICA on fMRI datasets. Neuroimage.

[b0335] Yeo B.T.T., Krienen F.M., Sepulcre J., Sabuncu M.R., Lashkari D., Hollinshead M. (2011). The organization of the human cerebral cortex estimated by intrinsic functional connectivity. J. Neurophysiol..

[b0340] Zhai T., Shao Y., Chen G., Ye E., Ma L., Wang L. (2015). Nature of functional links in valuation networks differentiates impulsive behaviors between abstinent heroin-dependent subjects and nondrug-using subjects. Neuroimage.

[b0345] Zhang, R., Geng, X., Lee, T.M.C. 2017. Large-scale functional neural network correlates of response inhibition: an fMRI meta-analysis. Brain Struct. Funct. [Internet]. 222(9):3973–3990. Available from: https://pubmed.ncbi.nlm.nih.gov/28551777. 10.1007/s00429-017-1443-x PMC5686258 28551777

