# Supplementary material for: Testing the triple network model of psychopathology in a transdiagnostic neurodevelopmental cohort
Source: Neuroimage Clin. 2023 Nov 9;40:103539. doi: 10.1016/j.nicl.2023.103539 (PMC10709083; doi:10.1016/j.nicl.2023.103539)
Supplement: Supplementary data 1 [file mmc1.docx]

**Table S1.**

**Triple Network Connectivity and Neurodevelopmental Difficulties in At-Risk Children**

|  | **Hyperactivity/Impulsivity** | | | | | | | **Inattention** | | | | | | | | |
| --- | --- | --- | --- | --- | --- | --- | --- | --- | --- | --- | --- | --- | --- | --- | --- | --- |
|  | *B* | | *SE* | | 95% CIs | *p* | | *B* | | *SE* | | 95% CIs | | *p* | |  |
| **SN - CEN** | 0.21 | 0.08 | | 0.06:0.36 | | 0.006** | 0.02 | | 0.08 | | -0.14:0.18 | | 0.784 | |  |  |
| **SN - DMN** | 0.2 | 0.08 | | 0.04:0.36 | | 0.013* | 0.05 | | 0.09 | | -0.11:0.22 | | 0.525 | |  |  |
| **CEN - DMN** | 0.16 | 0.08 | | 0.01:0.32 | | 0.042* | 0.06 | | 0.08 | | -0.11:0.23 | | 0.484 | |  |  |

*Note.* Standardised beta coefficients are reported with Standard Errors (SE) and 95% Confidence Intervals (CIs), controlling for age, age^2^, gender, and in-scanner motion. Salience Network (SN), Central Executive Network (CEN), and Default Mode Network (DMN). **p* < 0.05, ***p* < 0.01

**Table S2.**

**Triple Network Connectivity and Neurodevelopmental Difficulties in Comparison Children**

|  | **Hyperactivity/Impulsivity** | | | | **Inattention** | | | | | |
| --- | --- | --- | --- | --- | --- | --- | --- | --- | --- | --- |
|  | *B* | *SE* | 95% CIs | *p* | | *B* | *SE* | 95% CIs | *p* |  |
| **SN - CEN** | 0.04 | 0.14 | -0.25:0.33 | 0.785 | | -0.02 | 0.14 | -0.30:0.26 | 0.9 |  |
| **SN - DMN** | 0.07 | 0.16 | -0.25:0.38 | 0.669 | | 0.06 | 0.15 | -0.24:0.36 | 0.698 |  |
| **CEN - DMN** | -0.54 | 0.12 | -0.79:-0.29 | 6.7E-5*** | | -0.12 | 0.14 | -0.40:0.17 | 0.413 |  |

*Note.* Standardised beta coefficients are reported with Standard Errors (SE) and 95% Confidence Intervals (CIs), controlling for age, age^2^, gender, and in-scanner motion. Salience Network (SN), Central Executive Network (CEN), and Default Mode Network (DMN). ****p* < 0.001

**Table S3.**

**Relationship between FC and hyperactivity/impulsivity in the at-risk sample split by diagnostic status**

|  |  | **Hyperactivity/Impulsivity** | | | |
| --- | --- | --- | --- | --- | --- |
|  |  | *B* | *SE* | 95% CIs | *p* |
| **CEN-SN** | **At-Risk with ADHD** | .439 | .007 | -.001:.027 | .060 |
|  | **At-Risk without ADHD** | .183 | .003 | .001:.012 | .030* |
|  | **At-Risk with Diagnosis** | .272 | .004 | .000:.015 | .046* |
|  | **At-Risk w/o Diagnosis** | .211 | .004 | .001:.015 | .027* |
| **DMN-SN** | **At-Risk with ADHD** | .167 | .010 | -.013:.027 | .473 |
|  | **At-Risk without ADHD** | .146 | .004 | -.001:.014 | .086 |
|  | **At-Risk with Diagnosis** | .377 | .005 | .005:.026 | .005** |
|  | **At-Risk w/o Diagnosis** | .145 | .004 | -.002:.015 | .128 |
| **CEN-DMN** | **At-Risk with ADHD** | .262 | .008 | -.007:.025 | .270 |
|  | **At-Risk without ADHD** | .151 | .003 | -.001:.011 | .081 |
|  | **At-Risk with Diagnosis** | .371 | .004 | .003:.011 | .009** |
|  | **At-Risk w/o Diagnosis** | .083 | .003 | -.004:.010 | .392 |

*Note.* Standardised beta coefficients are reported with Standard Errors (SE) and 95% Confidence Intervals (CIs), controlling for age, age^2^, gender, and in-scanner motion. Salience Network (SN), Central Executive Network (CEN), and Default Mode Network (DMN). **p* < 0.05, ***p* < 0.01

|  | **Hyperactivity/Impulsivity** | | | | **Inattention** | | | | | |
| --- | --- | --- | --- | --- | --- | --- | --- | --- | --- | --- |
|  | *B* | *SE* | 95% CIs | *p* | | *B* | *SE* | 95% CIs | *p* |  |
| **Ventral SN - CEN** | 0.03 | 0.08 | -0.13:0.19 | 0.723 | | 0.02 | 0.09 | -0.15:0.20 | 0.798 |  |
| **Ventral SN - DMN** | 0.2 | 0.08 | 0.05:0.36 | 0.009** | | 0.03 | 0.08 | -0.13:0.19 | 0.718 |  |
| **Dorsal SN - CEN** | -0.07 | 0.08 | -0.23:0.09 | 0.373 | | 0.14 | 0.08 | -0.04:0.29 | 0.128 |  |
| **Dorsal SN - DMN** | 0.15 | 0.08 | 0.00:0.31 | 0.05* | | -0.01 | 0.08 | -0.17:0.15 | 0.902 |  |
| **CEN - DMN** | 0.21 | 0.08 | 0.05:0.36 | 0.009** | | 0.11 | 0.08 | -0.05:0.27 | 0.189 |  |

**Table S4.**

**Adult Network Connectivity and Neurodevelopmental Difficulties in At-Risk Children**

*Note.* Standardised beta coefficients are reported with Standard Errors (SE) and 95% Confidence Intervals (CIs), controlling for age, age^2^, gender, and in-scanner motion. Salience Network (SN), Central Executive Network (CEN), and Default Mode Network (DMN). **p* < 0.05, ***p* < 0.01

**Table S5.**

**Adult Network Connectivity and Neurodevelopmental Difficulties in Comparison Children**

|  | **Hyperactivity/Impulsivity** | | | | **Inattention** | | | | | |
| --- | --- | --- | --- | --- | --- | --- | --- | --- | --- | --- |
|  | *B* | *SE* | 95% CIs | *p* | | *B* | *SE* | 95% CIs | *p* |  |
| **Ventral SN - CEN** | 0.15 | 0.15 | -0.15:0.46 | 0.317 | | 0 | 0.16 | -0.31:0.31 | 0.998 |  |
| **Ventral SN - DMN** | -0.16 | 0.14 | -0.45:0.13 | 0.281 | | -0.13 | 0.14 | -0.41:0.16 | 0.385 |  |
| **Dorsal SN - CEN** | 0.14 | 0.15 | -0.15:0.44 | 0.334 | | 0.07 | 0.14 | -0.22:0.36 | 0.63 |  |
| **Dorsal SN - DMN** | -0.09 | 0.14 | -0.39:0.20 | 0.515 | | -0.09 | 0.14 | -0.38:0.19 | 0.506 |  |
| **CEN - DMN** | -0.22 | 0.14 | -0.50:0.05 | 0.11 | | -0.18 | 0.14 | -0.45:0.10 | 0.196 |  |

*Note.* Standardised beta coefficients are reported with Standard Errors (SE) and 95% Confidence Intervals (CIs), controlling for age, age^2^, gender, and in-scanner motion. Salience Network (SN), Central Executive Network (CEN), and Default Mode Network (DMN).

**Table S6.**

**Network Connectivity Differences between the Samples**

|  | At-Risk | Comparison | t-test | | | Regression | | | |
| --- | --- | --- | --- | --- | --- | --- | --- | --- | --- |
|  | *M* (*SD*) | *M* (*SD*) | *t* | *D* | *p* | *β* | *SE* | CI | *p* |
| **SN - CEN** | 0.11 (0.17) | 0.06 (0.16) | 2.1 | -0.32 | 0.036* | 0.19 | 0.15 | -0.11:0.49 | 0.22 |
| **SN - DMN** | 0.17 (0.19) | 0.19 (0.21) | -0.73 | 0.11 | 0.465 | -0.08 | 0.16 | -0.40:0.23 | 0.599 |
| **CEN - DMN** | 0.19 (0.15) | 0.17 (0.16) | 0.78 | -0.12 | 0.434 | 0.02 | 0.16 | -0.28:0.33 | 0.874 |

*Note.* Standardised beta coefficients are reported with Standard Errors (SE) and 95% Confidence Intervals (CIs), controlling for age, age^2^, gender, and in-scanner motion. Cohen’s D (*D*), Salience Network (SN), Central Executive Network (CEN), Default Mode Network (DMN). **p* < 0.05

**Table S7.**

**Network Connectivity Differences in Children Diagnosed with Combined Type ADHD**

|  | ADHD | Comparison | t-test | | | Regression | | | |
| --- | --- | --- | --- | --- | --- | --- | --- | --- | --- |
|  | *M* (*SD*) | *M* (*SD*) | *t* | *D* | *p* | *β* | *SE* | CI | *p* |
| **SN - CEN** | 0.16 (0.13) | 0.06 (0.16) | 3.08 | 0.7 | 0.003** | 0.56 | 0.24 | 0.08:-1.04 | 0.023* |
| **SN - DMN** | 0.23 (0.17) | 0.19 (0.21) | 0.78 | 0.18 | 0.44 | 0.15 | 0.26 | -0.36:0.67 | 0.554 |
| **CEN - DMN** | 0.24 (0.08) | 0.18 (0.16) | 1.73 | 0.45 | 0.087 | 0.18 | 0.27 | -0.36:0.71 | 0.512 |

*Note.* Standardised beta coefficients are reported with Standard Errors (SE) and 95% Confidence Intervals (CIs), controlling for age, age^2^, gender, and in-scanner motion. Attention Deficit Hyperactivity Disorder (ADHD), Cohen’s D (*D*), Salience Network (SN), Central Executive Network (CEN), Default Mode Network (DMN). **p* < 0.05, ***p* < 0.01

**Table S8.**

**Regional Salience - Central Executive Network Connectivity and Neurodevelopmental Difficulties in At-Risk Children**

|  | **Hyperactivity/Impulsivity** | | | | **Inattention** | | | | | |
| --- | --- | --- | --- | --- | --- | --- | --- | --- | --- | --- |
|  | *B* | *SE* | 95% CIs | *p* | | *B* | *SE* | 95% CIs | *p* |  |
| L SN dAI - L CEN lPFC | 0.06 | 0.08 | -0.10:0.22 | 0.831 | | 0.09 | 0.08 | -0.07:0.26 | 0.787 |  |
| L SN dAI - L CEN IPS | 0.02 | 0.08 | -0.14:0.18 | 0.919 | | 0.01 | 0.08 | -0.15:0.18 | 0.994 |  |
| L SN dAI - R CEN lPFC | 0.03 | 0.08 | -0.13:0.19 | 0.868 | | 0 | 0.08 | -0.16:0.16 | 0.994 |  |
| L SN dAI - R CEN IPS | -0.01 | 0.08 | -0.17:0.15 | 0.919 | | 0.06 | 0.09 | -0.11:0.23 | 0.787 |  |
| L SN dAC - L CEN lPFC | -0.03 | 0.08 | -0.19:0.13 | 0.868 | | 0.05 | 0.08 | -0.12:0.21 | 0.787 |  |
| L SN dAC - L CEN IPS | 0.06 | 0.08 | -0.11:0.22 | 0.831 | | 0.11 | 0.08 | -0.06:0.28 | 0.787 |  |
| L SN dAC - R CEN lPFC | 0.04 | 0.08 | -0.12:0.20 | 0.868 | | 0.11 | 0.08 | -0.06:0.28 | 0.787 |  |
| L SN dAC - R CEN IPS | -0.06 | 0.08 | -0.22:0.10 | 0.831 | | 0.15 | 0.08 | -0.01:0.31 | 0.787 |  |
| L SN vAI - L CEN lPFC | 0.11 | 0.08 | -0.04:0.27 | 0.689 | | -0.14 | 0.08 | -0.30:0.03 | 0.787 |  |
| L SN vAI - L CEN IPS | 0.07 | 0.08 | -0.09:0.23 | 0.831 | | -0.08 | 0.09 | -0.25:0.09 | 0.787 |  |
| L SN vAI - R CEN lPFC | 0.12 | 0.08 | -0.04:0.28 | 0.689 | | 0 | 0.08 | -0.16:0.16 | 0.994 |  |
| L SN vAI - R CEN IPS | 0.11 | 0.08 | -0.05:0.27 | 0.689 | | -0.1 | 0.08 | -0.27:0.07 | 0.787 |  |
| R SN dAI - L CEN lPFC | 0.2 | 0.08 | 0.04:0.36 | 0.394 | | 0.01 | 0.09 | -0.16:0.18 | 0.994 |  |
| R SN dAI - L CEN IPS | -0.03 | 0.08 | -0.20:0.14 | 0.868 | | 0.08 | 0.09 | -0.09:0.25 | 0.787 |  |
| R SN dAI - R CEN lPFC | -0.01 | 0.08 | -0.17:0.15 | 0.919 | | -0.05 | 0.08 | -0.22:0.11 | 0.787 |  |
| R SN dAI - R CEN IPS | -0.07 | 0.08 | -0.24:0.09 | 0.831 | | 0.05 | 0.08 | -0.11:0.22 | 0.787 |  |
| R SN dAC - L CEN lPFC | -0.04 | 0.08 | -0.20:0.12 | 0.868 | | 0.06 | 0.09 | -0.11:0.23 | 0.787 |  |
| R SN dAC - L CEN IPS | 0.01 | 0.08 | -0.15:0.17 | 0.919 | | 0.09 | 0.08 | -0.07:0.26 | 0.787 |  |
| R SN dAC - R CEN lPFC | 0.03 | 0.08 | -0.13:0.19 | 0.868 | | 0.06 | 0.08 | -0.10:0.23 | 0.787 |  |
| R SN dAC - R CEN IPS | -0.06 | 0.08 | -0.22:0.11 | 0.831 | | 0.05 | 0.09 | -0.12:0.22 | 0.787 |  |
| R SN vAI - L CEN lPFC | 0.1 | 0.08 | -0.06:0.25 | 0.725 | | 0.01 | 0.08 | -0.16:0.17 | 0.994 |  |
| R SN vAI - L CEN IPS | 0.11 | 0.08 | -0.05:0.27 | 0.689 | | 0.08 | 0.08 | -0.09:0.24 | 0.787 |  |
| R SN vAI - R CEN lPFC | 0.11 | 0.08 | -0.05:0.27 | 0.689 | | 0.05 | 0.08 | -0.12:0.21 | 0.787 |  |
| R SN vAI - R CEN IPS | 0.09 | 0.08 | -0.06:0.25 | 0.725 | | 0.04 | 0.08 | -0.13:0.21 | 0.792 |  |

*Note.* Standardised beta coefficients are reported with Standard Errors (SE) and 95% Confidence Intervals (CIs), controlling for age, age^2^, gender, and in-scanner motion. Salience Network (SN), Central Executive Network (CEN), Left (L), Right (R), dorsal Anterior Insula (dAI), dorsal Anterior Cingulate (dAC), ventral Anterior Insula (vAI), lateral Prefrontal Cortex (lPFC), and Intraparietal Sulcus (IPS).

**Table S9.**

**Regional Salience - Central Executive Network Connectivity and Neurodevelopmental Difficulties in Comparison Children**

*Note.* Standardised beta coefficients are reported with Standard Errors (SE) and 95% Confidence Intervals (CIs), controlling for age, age^2^, gender, and in-scanner motion. Left (L), Right (R), Salience Network (SN), Central Executive Network (CEN), dorsal Anterior Insula (dAI), dorsal Anterior Cingulate (dAC), ventral Anterior Insula (vAI), lateral Prefrontal Cortex (lPFC), and Intraparietal Sulcus (IPS).

|  | **Hyperactivity/Impulsivity** | | | | **Inattention** | | | | | |
| --- | --- | --- | --- | --- | --- | --- | --- | --- | --- | --- |
|  | *B* | *SE* | 95% CIs | *p* | | *B* | *SE* | 95% CIs | *p* |  |
| L SN dAI - L CEN lPFC | 0.26 | 0.14 | -0.02:0.55 | 0.324 | | 0.1 | 0.14 | -0.19:0.39 | 0.755 |  |
| L SN dAI - L CEN IPS | 0.14 | 0.15 | -0.16:0.43 | 0.751 | | 0.11 | 0.14 | -0.18:0.39 | 0.755 |  |
| L SN dAI - R CEN lPFC | 0.08 | 0.14 | -0.21:0.36 | 0.834 | | 0.09 | 0.14 | -0.18:0.37 | 0.755 |  |
| L SN dAI - R CEN IPS | 0.06 | 0.15 | -0.25:0.37 | 0.877 | | 0.17 | 0.15 | -0.13:0.48 | 0.755 |  |
| L SN dAC - L CEN lPFC | -0.18 | 0.15 | -0.48:0.12 | 0.685 | | -0.03 | 0.15 | -0.34:0.28 | 0.963 |  |
| L SN dAC - L CEN IPS | 0.02 | 0.15 | -0.28:0.32 | 0.961 | | 0.09 | 0.15 | -0.21:0.39 | 0.755 |  |
| L SN dAC - R CEN lPFC | -0.02 | 0.15 | -0.31:0.27 | 0.961 | | -0.01 | 0.15 | -0.30:0.28 | 0.996 |  |
| L SN dAC - R CEN IPS | -0.01 | 0.15 | -0.31:0.30 | 0.961 | | 0.18 | 0.14 | -0.11:0.47 | 0.755 |  |
| L SN vAI - L CEN lPFC | 0.03 | 0.14 | -0.26:0.32 | 0.961 | | 0.08 | 0.14 | -0.20:0.37 | 0.755 |  |
| L SN vAI - L CEN IPS | -0.11 | 0.14 | -0.40:0.18 | 0.821 | | -0.13 | 0.14 | -0.41:0.15 | 0.755 |  |
| L SN vAI - R CEN lPFC | -0.26 | 0.15 | -0.55:0.03 | 0.324 | | -0.24 | 0.15 | -0.53:0.05 | 0.755 |  |
| L SN vAI - R CEN IPS | -0.29 | 0.15 | -0.59:0.01 | 0.324 | | -0.09 | 0.15 | -0.39:0.20 | 0.755 |  |
| R SN dAI - L CEN lPFC | 0.34 | 0.14 | 0.05:0.62 | 0.324 | | 0.1 | 0.14 | -0.19:0.39 | 0.755 |  |
| R SN dAI - L CEN IPS | 0.1 | 0.15 | -0.21:0.40 | 0.821 | | 0 | 0.15 | -0.29:0.30 | 0.996 |  |
| R SN dAI - R CEN lPFC | 0.13 | 0.15 | -0.16:0.43 | 0.751 | | 0.09 | 0.14 | -0.20:0.38 | 0.755 |  |
| R SN dAI - R CEN IPS | 0.16 | 0.15 | -0.14:0.45 | 0.751 | | 0.07 | 0.15 | -0.23:0.37 | 0.806 |  |
| R SN dAC - L CEN lPFC | -0.01 | 0.15 | -0.32:0.29 | 0.961 | | -0.15 | 0.15 | -0.45:0.15 | 0.755 |  |
| R SN dAC - L CEN IPS | 0.1 | 0.16 | -0.23:0.43 | 0.821 | | -0.15 | 0.16 | -0.47:0.16 | 0.755 |  |
| R SN dAC - R CEN lPFC | -0.06 | 0.15 | -0.37:0.25 | 0.877 | | -0.13 | 0.15 | -0.43:0.16 | 0.755 |  |
| R SN dAC - R CEN IPS | 0.19 | 0.15 | -0.11:0.50 | 0.685 | | 0.02 | 0.15 | -0.27:0.31 | 0.963 |  |
| R SN vAI - L CEN lPFC | 0.26 | 0.14 | -0.03:0.54 | 0.324 | | 0.15 | 0.14 | -0.14:0.43 | 0.755 |  |
| R SN vAI - L CEN IPS | 0.31 | 0.13 | 0.04:0.58 | 0.324 | | -0.04 | 0.14 | -0.31:0.24 | 0.959 |  |
| R SN vAI - R CEN lPFC | -0.15 | 0.17 | -0.49:0.19 | 0.751 | | -0.19 | 0.15 | -0.49:0.12 | 0.755 |  |
| R SN vAI - R CEN IPS | 0.09 | 0.15 | -0.21:0.40 | 0.821 | | -0.09 | 0.15 | -0.39:0.20 | 0.755 |  |

**Table S10.**

**Regional Salience – Default Mode Network Connectivity and Neurodevelopmental Difficulties in At-Risk Children**

|  | **Hyperactivity/Impulsivity** | | | | **Inattention** | | | | | |
| --- | --- | --- | --- | --- | --- | --- | --- | --- | --- | --- |
|  | *B* | *SE* | 95% CIs | *p* | | *B* | *SE* | 95% CIs | *p* |  |
| L SN dAI - L DMN mPFC | -0.02 | 0.08 | -0.17:0.13 | 0.838 | | 0.12 | 0.08 | -0.04:0.27 | 0.834 |  |
| L SN dAI - L DMN PCC | 0.07 | 0.08 | -0.08:0.23 | 0.482 | | 0.02 | 0.08 | -0.14:0.18 | 0.856 |  |
| L SN dAI - R DMN mPFC | 0.05 | 0.08 | -0.10:0.20 | 0.593 | | 0.14 | 0.08 | -0.01:0.30 | 0.834 |  |
| L SN dAI - R DMN PCC | 0.1 | 0.08 | -0.06:0.26 | 0.412 | | 0.04 | 0.08 | -0.13:0.20 | 0.834 |  |
| L SN dAC - L DMN mPFC | 0.08 | 0.08 | -0.08:0.24 | 0.482 | | 0.03 | 0.09 | -0.14:0.20 | 0.852 |  |
| L SN dAC - L DMN PCC | 0.18 | 0.08 | 0.02:0.34 | 0.125 | | -0.07 | 0.08 | -0.24:0.09 | 0.834 |  |
| L SN dAC - R DMN mPFC | 0.12 | 0.08 | -0.04:0.28 | 0.351 | | -0.08 | 0.08 | -0.25:0.08 | 0.834 |  |
| L SN dAC - R DMN PCC | 0.16 | 0.08 | 0.00:0.32 | 0.149 | | -0.09 | 0.08 | -0.26:0.07 | 0.834 |  |
| L SN vAI - L DMN mPFC | 0 | 0.08 | -0.16:0.17 | 0.985 | | 0.02 | 0.08 | -0.15:0.19 | 0.856 |  |
| L SN vAI - L DMN PCC | 0.17 | 0.08 | 0.01:0.32 | 0.139 | | 0.05 | 0.08 | -0.11:0.21 | 0.834 |  |
| L SN vAI - R DMN mPFC | 0.06 | 0.08 | -0.10:0.22 | 0.565 | | 0.04 | 0.08 | -0.13:0.21 | 0.834 |  |
| L SN vAI - R DMN PCC | 0.13 | 0.08 | -0.03:0.29 | 0.337 | | 0.06 | 0.08 | -0.11:0.23 | 0.834 |  |
| R SN dAI - L DMN mPFC | 0 | 0.08 | -0.16:0.15 | 0.985 | | 0.06 | 0.08 | -0.10:0.22 | 0.834 |  |
| R SN dAI - L DMN PCC | 0.12 | 0.08 | -0.04:0.28 | 0.337 | | -0.05 | 0.08 | -0.22:0.11 | 0.834 |  |
| R SN dAI - R DMN mPFC | 0.07 | 0.08 | -0.09:0.23 | 0.499 | | 0.08 | 0.08 | -0.08:0.25 | 0.834 |  |
| R SN dAI - R DMN PCC | 0.07 | 0.08 | -0.08:0.23 | 0.482 | | -0.07 | 0.08 | -0.23:0.09 | 0.834 |  |
| R SN dAC - L DMN mPFC | 0.09 | 0.08 | -0.07:0.24 | 0.435 | | 0.09 | 0.08 | -0.07:0.25 | 0.834 |  |
| R SN dAC - L DMN PCC | 0.26 | 0.08 | 0.10:0.41 | 0.028* | | 0.05 | 0.08 | -0.12:0.21 | 0.834 |  |
| R SN dAC - R DMN mPFC | 0.09 | 0.08 | -0.06:0.25 | 0.412 | | 0.05 | 0.08 | -0.12:0.21 | 0.834 |  |
| R SN dAC - R DMN PCC | 0.22 | 0.08 | 0.06:0.37 | 0.061 | | -0.04 | 0.08 | -0.20:0.13 | 0.834 |  |
| R SN vAI - L DMN mPFC | 0.1 | 0.08 | -0.06:0.26 | 0.412 | | 0.03 | 0.08 | -0.14:0.19 | 0.856 |  |
| R SN vAI - L DMN PCC | 0.21 | 0.08 | 0.05:0.37 | 0.061^+^ | | 0.05 | 0.09 | -0.12:0.22 | 0.834 |  |
| R SN vAI - R DMN mPFC | 0.1 | 0.08 | -0.05:0.26 | 0.412 | | -0.02 | 0.08 | -0.18:0.15 | 0.856 |  |
| R SN vAI - R DMN PCC | 0.21 | 0.08 | 0.06:0.36 | 0.061^+^ | | 0.04 | 0.08 | -0.12:0.20 | 0.834 |  |

*Note.* Standardised beta coefficients are reported with Standard Errors (SE) and 95% Confidence Intervals (CIs), controlling for age, age^2^, gender, and in-scanner motion. Left (L), Right (R), Salience Network (SN), Default Mode Network (DMN), dorsal Anterior Insula (dAI), dorsal Anterior Cingulate (dAC), ventral Anterior Insula (vAI), medial Prefrontal Cortex (mPFC), and Posterior Cingulate Cortex (PCC). ^+^p < 0.062, *p < 0.05

**Table S11.**

**Regional Salience – Default Mode Network Connectivity and Neurodevelopmental Difficulties in Comparison**

|  | **Hyperactivity/Impulsivity** | | | | **Inattention** | | | | | |
| --- | --- | --- | --- | --- | --- | --- | --- | --- | --- | --- |
|  | *B* | *SE* | 95% CIs | *p* | | *B* | *SE* | 95% CIs | *p* |  |
| L SN dAI - L DMN mPFC | -0.12 | 0.15 | -0.41:0.17 | 0.879 | | -0.05 | 0.14 | -0.34:0.24 | 0.949 |  |
| L SN dAI - L DMN PCC | 0.11 | 0.15 | -0.18:0.41 | 0.879 | | 0.11 | 0.14 | -0.18:0.40 | 0.949 |  |
| L SN dAI - R DMN mPFC | -0.18 | 0.15 | -0.48:0.13 | 0.879 | | -0.3 | 0.15 | -0.59:-0.00 | 0.949 |  |
| L SN dAI - R DMN PCC | 0.06 | 0.14 | -0.23:0.36 | 0.879 | | 0.13 | 0.14 | -0.15:0.41 | 0.949 |  |
| L SN dAC - L DMN mPFC | -0.08 | 0.15 | -0.37:0.22 | 0.879 | | -0.2 | 0.14 | -0.49:0.08 | 0.949 |  |
| L SN dAC - L DMN PCC | -0.09 | 0.14 | -0.38:0.20 | 0.879 | | -0.05 | 0.14 | -0.33:0.24 | 0.949 |  |
| L SN dAC - R DMN mPFC | -0.11 | 0.16 | -0.42:0.20 | 0.879 | | -0.11 | 0.15 | -0.41:0.20 | 0.949 |  |
| L SN dAC - R DMN PCC | -0.02 | 0.14 | -0.30:0.27 | 0.946 | | -0.03 | 0.14 | -0.31:0.25 | 0.97 |  |
| L SN vAI - L DMN mPFC | 0.07 | 0.15 | -0.23:0.37 | 0.879 | | 0.01 | 0.15 | -0.29:0.30 | 0.973 |  |
| L SN vAI - L DMN PCC | 0.09 | 0.15 | -0.22:0.39 | 0.879 | | -0.06 | 0.15 | -0.37:0.24 | 0.949 |  |
| L SN vAI - R DMN mPFC | -0.05 | 0.14 | -0.33:0.24 | 0.943 | | 0.06 | 0.15 | -0.23:0.35 | 0.949 |  |
| L SN vAI - R DMN PCC | -0.01 | 0.15 | -0.31:0.29 | 0.946 | | -0.07 | 0.14 | -0.36:0.22 | 0.949 |  |
| R SN dAI - L DMN mPFC | -0.01 | 0.15 | -0.32:0.30 | 0.946 | | -0.05 | 0.15 | -0.35:0.26 | 0.949 |  |
| R SN dAI - L DMN PCC | 0.09 | 0.15 | -0.20:0.39 | 0.879 | | 0.07 | 0.15 | -0.22:0.36 | 0.949 |  |
| R SN dAI - R DMN mPFC | 0.03 | 0.15 | -0.28:0.34 | 0.946 | | -0.1 | 0.15 | -0.40:0.20 | 0.949 |  |
| R SN dAI - R DMN PCC | 0.12 | 0.15 | -0.19:0.42 | 0.879 | | 0.06 | 0.15 | -0.24:0.35 | 0.949 |  |
| R SN dAC - L DMN mPFC | -0.18 | 0.14 | -0.47:0.11 | 0.879 | | -0.11 | 0.14 | -0.40:0.17 | 0.949 |  |
| R SN dAC - L DMN PCC | -0.09 | 0.14 | -0.38:0.19 | 0.879 | | 0.06 | 0.14 | -0.22:0.33 | 0.949 |  |
| R SN dAC - R DMN mPFC | -0.08 | 0.15 | -0.37:0.22 | 0.879 | | -0.09 | 0.14 | -0.38:0.19 | 0.949 |  |
| R SN dAC - R DMN PCC | -0.03 | 0.14 | -0.31:0.24 | 0.946 | | -0.01 | 0.13 | -0.28:0.25 | 0.973 |  |
| R SN vAI - L DMN mPFC | -0.27 | 0.14 | -0.55:0.02 | 0.795 | | 0.02 | 0.14 | -0.27:0.31 | 0.973 |  |
| R SN vAI - L DMN PCC | -0.23 | 0.15 | -0.54:0.07 | 0.879 | | -0.13 | 0.15 | -0.44:0.18 | 0.949 |  |
| R SN vAI - R DMN mPFC | -0.35 | 0.14 | -0.64:-0.06 | 0.421 | | -0.01 | 0.15 | -0.31:0.29 | 0.973 |  |
| R SN vAI - R DMN PCC | -0.14 | 0.15 | -0.45:0.17 | 0.879 | | -0.22 | 0.15 | -0.52:0.08 | 0.949 |  |

*Note.* Standardised beta coefficients are reported with Standard Errors (SE) and 95% Confidence Intervals (CIs), controlling for age, age^2^, gender, and in-scanner motion. Left (L), Right (R), Salience Network (SN), Default Mode Network (DMN), dorsal Anterior Insula (dAI), dorsal Anterior Cingulate (dAC), ventral Anterior Insula (vAI), medial Prefrontal Cortex (mPFC), and Posterior Cingulate Cortex (PCC).

**Table S12.**

**Regional Central Executive – Default Mode Network Connectivity and Neurodevelopmental Difficulties in At-Risk Children**

|  | **Hyperactivity/Impulsivity** | | | | **Inattention** | | | | | |
| --- | --- | --- | --- | --- | --- | --- | --- | --- | --- | --- |
|  | *B* | *SE* | 95% CIs | *p* | | *B* | *SE* | 95% CIs | *p* |  |
| L DMN mPFC - L CEN lPFC | 0.19 | 0.08 | 0.03:0.35 | 0.072^+^ | | 0.05 | 0.08 | -0.12:0.21 | 0.668 |  |
| L DMN mPFC - L CEN IPS | 0.18 | 0.08 | 0.03:0.33 | 0.072^+^ | | 0.11 | 0.08 | -0.05:0.27 | 0.58 |  |
| L DMN mPFC - R CEN lPFC | 0.29 | 0.08 | 0.14:0.43 | 0.003** | | 0.14 | 0.08 | -0.02:0.29 | 0.478 |  |
| L DMN mPFC - R CEN IPS | 0.06 | 0.08 | -0.10:0.22 | 0.613 | | 0.1 | 0.08 | -0.07:0.26 | 0.58 |  |
| L DMN PCC - L CEN lPFC | 0.07 | 0.08 | -0.09:0.23 | 0.597 | | 0.07 | 0.09 | -0.10:0.24 | 0.616 |  |
| L DMN PCC - L CEN IPS | -0.01 | 0.08 | -0.17:0.15 | 0.958 | | 0.04 | 0.08 | -0.13:0.21 | 0.68 |  |
| L DMN PCC - R CEN lPFC | 0.16 | 0.08 | 0.01:0.32 | 0.108 | | 0.06 | 0.08 | -0.11:0.22 | 0.616 |  |
| L DMN PCC - R CEN IPS | 0.11 | 0.08 | -0.05:0.27 | 0.367 | | -0.06 | 0.09 | -0.23:0.11 | 0.616 |  |
| R DMN mPFC - L CEN lPFC | 0.18 | 0.08 | 0.03:0.34 | 0.072^+^ | | 0.02 | 0.08 | -0.14:0.19 | 0.778 |  |
| R DMN mPFC - L CEN IPS | 0.14 | 0.08 | -0.02:0.29 | 0.184 | | 0.14 | 0.08 | -0.02:0.30 | 0.478 |  |
| R DMN mPFC - R CEN lPFC | 0.25 | 0.08 | 0.10:0.40 | 0.008** | | 0.07 | 0.08 | -0.09:0.24 | 0.58 |  |
| R DMN mPFC - R CEN IPS | 0.03 | 0.08 | -0.13:0.19 | 0.886 | | 0.1 | 0.08 | -0.07:0.26 | 0.58 |  |
| R DMN PCC - L CEN lPFC | 0.07 | 0.08 | -0.10:0.23 | 0.606 | | 0.1 | 0.08 | -0.07:0.27 | 0.58 |  |
| R DMN PCC - L CEN IPS | 0.02 | 0.08 | -0.14:0.18 | 0.929 | | 0.08 | 0.08 | -0.09:0.24 | 0.58 |  |
| R DMN PCC - R CEN lPFC | 0.08 | 0.08 | -0.08:0.24 | 0.543 | | 0.09 | 0.08 | -0.08:0.25 | 0.58 |  |
| R DMN PCC - R CEN IPS | 0 | 0.08 | -0.16:0.17 | 0.959 | | -0.16 | 0.08 | -0.33:0.00 | 0.478 |  |

*Note.* Standardised beta coefficients are reported with Standard Errors (SE) and 95% Confidence Intervals (CIs), controlling for age, age^2^, gender, and in-scanner motion. Left (L), Right (R), Central Executive Network (CEN), Default Mode Network (DMN), lateral Prefrontal Cortex (lPFC), Intraparietal Sulcus (IPS), medial Prefrontal Cortex (mPFC), and Posterior Cingulate Cortex (PCC). ^+^*p* < 0.073, **p* < 0.05, **p* < 0.01

**Table S13.**

**Regional Central Executive – Default Mode Network Connectivity and Neurodevelopmental Difficulties in Comparison Children**

|  | **Hyperactivity/Impulsivity** | | | | **Inattention** | | | | | |
| --- | --- | --- | --- | --- | --- | --- | --- | --- | --- | --- |
|  | *B* | *SE* | 95% CIs | *p* | | *B* | *SE* | 95% CIs | *p* |  |
| L DMN mPFC - L CEN lPFC | -0.23 | 0.13 | -0.50:0.04 | 0.344 | | -0.02 | 0.14 | -0.30:0.26 | 0.879 |  |
| L DMN mPFC - L CEN IPS | -0.19 | 0.14 | -0.48:0.09 | 0.344 | | -0.19 | 0.14 | -0.47:0.09 | 0.594 |  |
| L DMN mPFC - R CEN lPFC | -0.24 | 0.15 | -0.53:0.06 | 0.344 | | -0.16 | 0.15 | -0.46:0.13 | 0.594 |  |
| L DMN mPFC - R CEN IPS | -0.2 | 0.15 | -0.50:0.09 | 0.344 | | -0.04 | 0.14 | -0.33:0.25 | 0.835 |  |
| L DMN PCC - L CEN lPFC | -0.28 | 0.15 | -0.58:0.01 | 0.344 | | -0.11 | 0.16 | -0.43:0.20 | 0.666 |  |
| L DMN PCC - L CEN IPS | -0.08 | 0.14 | -0.37:0.20 | 0.637 | | -0.15 | 0.14 | -0.43:0.13 | 0.594 |  |
| L DMN PCC - R CEN lPFC | -0.24 | 0.14 | -0.52:0.04 | 0.344 | | -0.17 | 0.14 | -0.44:0.11 | 0.594 |  |
| L DMN PCC - R CEN IPS | -0.12 | 0.15 | -0.42:0.17 | 0.566 | | 0.07 | 0.14 | -0.22:0.36 | 0.767 |  |
| R DMN mPFC - L CEN lPFC | -0.15 | 0.13 | -0.41:0.11 | 0.401 | | -0.09 | 0.13 | -0.35:0.17 | 0.666 |  |
| R DMN mPFC - L CEN IPS | -0.02 | 0.15 | -0.33:0.28 | 0.88 | | -0.16 | 0.15 | -0.45:0.14 | 0.594 |  |
| R DMN mPFC - R CEN lPFC | -0.22 | 0.15 | -0.53:0.09 | 0.344 | | -0.28 | 0.15 | -0.58:0.02 | 0.594 |  |
| R DMN mPFC - R CEN IPS | -0.12 | 0.15 | -0.43:0.18 | 0.566 | | -0.21 | 0.15 | -0.50:0.08 | 0.594 |  |
| R DMN PCC - L CEN lPFC | -0.25 | 0.14 | -0.53:0.03 | 0.344 | | -0.12 | 0.14 | -0.41:0.16 | 0.666 |  |
| R DMN PCC - L CEN IPS | -0.04 | 0.14 | -0.32:0.24 | 0.852 | | -0.1 | 0.14 | -0.37:0.17 | 0.666 |  |
| R DMN PCC - R CEN lPFC | -0.18 | 0.14 | -0.46:0.10 | 0.344 | | -0.17 | 0.14 | -0.44:0.10 | 0.594 |  |
| R DMN PCC - R CEN IPS | -0.09 | 0.15 | -0.38:0.20 | 0.637 | | -0.04 | 0.14 | -0.33:0.25 | 0.835 |  |

*Note.* Standardised beta coefficients are reported with Standard Errors (SE) and 95% Confidence Intervals (CIs), controlling for age, age^2^, gender, and in-scanner motion. Left (L), Right (R), Central Executive Network (CEN), Default Mode Network (DMN), lateral Prefrontal Cortex (lPFC), Intraparietal Sulcus (IPS), medial Prefrontal Cortex (mPFC), and Posterior Cingulate Cortex (PCC).

**Table S14.**

**Within Salience Network Connectivity and Neurodevelopmental Difficulties in At-Risk Children**

|  | **Hyperactivity/Impulsivity** | | | | **Inattention** | | | | | |
| --- | --- | --- | --- | --- | --- | --- | --- | --- | --- | --- |
|  | *B* | *SE* | 95% CIs | *p* | | *B* | *SE* | 95% CIs | *p* |  |
| L SN dAI - L SN dAC | -0.14 | 0.08 | -0.29:0.02 | 0.418 | | 0.08 | 0.08 | -0.09:0.25 | 0.571 |  |
| L SN dAI - L SN vAI | -0.1 | 0.08 | -0.26:0.06 | 0.46 | | -0.02 | 0.08 | -0.18:0.14 | 0.878 |  |
| L SN dAI - R SN AI | 0.01 | 0.08 | -0.15:0.17 | 0.894 | | 0.18 | 0.08 | 0.01:0.34 | 0.351 |  |
| L SN dAI - R SN dAC | -0.1 | 0.08 | -0.26:0.06 | 0.46 | | -0.01 | 0.09 | -0.18:0.16 | 0.878 |  |
| L SN dAC - L SN vAI | -0.01 | 0.08 | -0.17:0.15 | 0.894 | | -0.13 | 0.08 | -0.29:0.04 | 0.5 |  |
| L SN dAC - R SN AI | -0.09 | 0.08 | -0.24:0.07 | 0.46 | | 0.12 | 0.08 | -0.05:0.28 | 0.5 |  |
| L SN dAC - R SN dAC | -0.01 | 0.08 | -0.17:0.15 | 0.894 | | 0.03 | 0.08 | -0.14:0.20 | 0.878 |  |
| L SN vAI - R SN AI | -0.1 | 0.08 | -0.26:0.07 | 0.46 | | 0.09 | 0.09 | -0.08:0.25 | 0.571 |  |
| L SN vAI - R SN dAC | -0.08 | 0.08 | -0.24:0.08 | 0.46 | | -0.11 | 0.08 | -0.28:0.06 | 0.5 |  |
| R SN dAI - R SN dAC | -0.21 | 0.08 | -0.37:-0.05 | 0.088 | | 0.04 | 0.09 | -0.13:0.21 | 0.878 |  |

*Note.* Standardised beta coefficients are reported with Standard Errors (SE) and 95% Confidence Intervals (CIs), controlling for age, age^2^, gender, and in-scanner motion. Left (L), Right (R), Salience Network (SN), dorsal Anterior Insula (dAI), dorsal Anterior Cingulate (dAC), and ventral Anterior Insula (vAI).

**Table S15.**

|  | **Hyperactivity/Impulsivity** | | | | **Inattention** | | | | | |
| --- | --- | --- | --- | --- | --- | --- | --- | --- | --- | --- |
|  | *B* | *SE* | 95% CIs | *p* | | *B* | *SE* | 95% CIs | *p* |  |
| L SN dAI - L SN dAC | -0.08 | 0.15 | -0.39:0.22 | 0.922 | | -0.2 | 0.15 | -0.50:0.10 | 0.928 |  |
| L SN dAI - L SN vAI | -0.1 | 0.15 | -0.39:0.20 | 0.922 | | 0.07 | 0.15 | -0.22:0.37 | 0.928 |  |
| L SN dAI - R SN AI | -0.04 | 0.15 | -0.34:0.26 | 0.922 | | 0.06 | 0.14 | -0.23:0.35 | 0.928 |  |
| L SN dAI - R SN dAC | -0.07 | 0.15 | -0.38:0.23 | 0.922 | | 0.01 | 0.15 | -0.29:0.32 | 0.928 |  |
| L SN dAC - L SN vAI | 0.03 | 0.15 | -0.27:0.33 | 0.922 | | 0.04 | 0.15 | -0.26:0.33 | 0.928 |  |
| L SN dAC - R SN AI | 0.06 | 0.15 | -0.24:0.36 | 0.922 | | -0.02 | 0.15 | -0.32:0.27 | 0.928 |  |
| L SN dAC - R SN dAC | -0.19 | 0.15 | -0.50:0.12 | 0.922 | | 0.02 | 0.15 | -0.28:0.32 | 0.928 |  |
| L SN vAI - R SN AI | 0.01 | 0.15 | -0.28:0.31 | 0.932 | | -0.09 | 0.15 | -0.39:0.21 | 0.928 |  |
| L SN vAI - R SN dAC | -0.23 | 0.14 | -0.52:0.06 | 0.922 | | -0.28 | 0.14 | -0.56:0.00 | 0.537 |  |
| R SN dAI - R SN dAC | 0.04 | 0.15 | -0.27:0.34 | 0.922 | | -0.1 | 0.15 | -0.40:0.20 | 0.928 |  |

**Within Salience Network Connectivity and Neurodevelopmental Difficulties in Comparison Children**

*Note.* Standardised beta coefficients are reported with Standard Errors (SE) and 95% Confidence Intervals (CIs), controlling for age, age^2^, gender, and in-scanner motion. Left (L), Right (R), Salience Network (SN), dorsal Anterior Insula (dAI), dorsal Anterior Cingulate (dAC), and ventral Anterior Insula (vAI).

**Table S16.**

|  | **Hyperactivity/Impulsivity** | | | | **Inattention** | | | | | |
| --- | --- | --- | --- | --- | --- | --- | --- | --- | --- | --- |
|  | *B* | *SE* | 95% CIs | *p* | | *B* | *SE* | 95% CIs | *p* |  |
| L CEN lPFC - L CEN IPS | -0.07 | 0.08 | -0.23:0.09 | 0.468 | | 0.08 | 0.08 | -0.08:0.25 | 0.641 |  |
| L CEN lPFC - R CEN lPFC | 0.14 | 0.08 | -0.02:0.30 | 0.281 | | 0.07 | 0.09 | -0.10:0.24 | 0.641 |  |
| L CEN lPFC - R CEN IPS | 0.01 | 0.08 | -0.15:0.17 | 0.926 | | -0.01 | 0.08 | -0.18:0.16 | 0.998 |  |
| L CEN IPS - R CEN lPFC | -0.09 | 0.08 | -0.25:0.07 | 0.468 | | 0 | 0.09 | -0.17:0.17 | 0.998 |  |
| L CEN IPS - R CEN IPS | 0.07 | 0.08 | -0.09:0.23 | 0.468 | | -0.15 | 0.08 | -0.32:0.01 | 0.397 |  |
| R CEN lPFC - R CEN IPS | -0.22 | 0.08 | -0.38:-0.06 | 0.04* | | -0.09 | 0.09 | -0.26:0.08 | 0.641 |  |

**Within Central Executive Network Connectivity and Neurodevelopmental Difficulties in At-Risk Children**

*Note.* Standardised beta coefficients are reported with Standard Errors (SE) and 95% Confidence Intervals (CIs), controlling for age, age^2^, gender, and in-scanner motion. Left (L), Right (R), Central Executive Network (CEN), lateral Prefrontal Cortex (lPFC), and Intraparietal Sulcus (IPS). **p* < 0.05

**Table S17.**

|  | **Hyperactivity/Impulsivity** | | | | **Inattention** | | | | | |
| --- | --- | --- | --- | --- | --- | --- | --- | --- | --- | --- |
|  | *B* | *SE* | 95% CIs | *p* | | *B* | *SE* | 95% CIs | *p* |  |
| L CEN lPFC - L CEN IPS | -0.35 | 0.13 | -0.62:-0.09 | 0.046* | | -0.02 | 0.13 | -0.29:0.25 | 0.901 |  |
| L CEN lPFC - R CEN lPFC | 0.15 | 0.16 | -0.18:0.47 | 0.747 | | 0.05 | 0.16 | -0.27:0.37 | 0.901 |  |
| L CEN lPFC - R CEN IPS | -0.02 | 0.15 | -0.33:0.29 | 0.964 | | -0.02 | 0.15 | -0.31:0.28 | 0.901 |  |
| L CEN IPS - R CEN lPFC | 0.01 | 0.15 | -0.30:0.31 | 0.964 | | 0.04 | 0.15 | -0.27:0.34 | 0.901 |  |
| L CEN IPS - R CEN IPS | 0.06 | 0.15 | -0.24:0.35 | 0.964 | | -0.07 | 0.15 | -0.36:0.23 | 0.901 |  |
| R CEN lPFC - R CEN IPS | 0.36 | 0.14 | 0.07:0.64 | 0.046* | | 0.09 | 0.15 | -0.21:0.39 | 0.901 |  |

**Within Central Executive Network Connectivity and Neurodevelopmental Difficulties in Comparison Children**

*Note.* Standardised beta coefficients are reported with Standard Errors (SE) and 95% Confidence Intervals (CIs), controlling for age, age^2^, gender, and in-scanner motion. Left (L), Right (R), Central Executive Network (CEN), lateral Prefrontal Cortex (lPFC), and Intraparietal Sulcus (IPS). **p* < 0.05

**Table S18.**

|  | **Hyperactivity/Impulsivity** | | | | **Inattention** | | | | | |
| --- | --- | --- | --- | --- | --- | --- | --- | --- | --- | --- |
|  | *B* | *SE* | 95% CIs | *p* | | *B* | *SE* | 95% CIs | *p* |  |
| L DMN mPFC - L DMN PCC | 0.14 | 0.08 | -0.02:0.30 | 0.162 | | -0.1 | 0.08 | -0.26:0.07 | 0.514 |  |
| L DMN mPFC - R DMN mPFC | 0.03 | 0.08 | -0.13:0.19 | 0.82 | | 0.03 | 0.08 | -0.14:0.19 | 0.742 |  |
| L DMN mPFC - R DMN PCC | 0.17 | 0.08 | 0.01:0.33 | 0.102 | | -0.07 | 0.08 | -0.23:0.10 | 0.514 |  |
| L DMN PCC - R DMN mPFC | 0.22 | 0.08 | 0.06:0.38 | 0.042* | | -0.07 | 0.08 | -0.24:0.10 | 0.514 |  |
| L DMN PCC - R DMN PCC | 0.02 | 0.08 | -0.14:0.18 | 0.82 | | -0.11 | 0.08 | -0.28:0.05 | 0.514 |  |
| R DMN mPFC - R DMN PCC | 0.11 | 0.08 | -0.04:0.27 | 0.237 | | -0.08 | 0.08 | -0.24:0.08 | 0.514 |  |

**Within Default Mode Network Connectivity and Neurodevelopmental Difficulties in At-Risk Children**

*Note.* Standardised beta coefficients are reported with Standard Errors (SE) and 95% Confidence Intervals (CIs), controlling for age, age^2^, gender, and in-scanner motion. Left (L), Right (R), Default Mode Network (DMN), medial Prefrontal Cortex (mPFC), and Posterior Cingulate Cortex (PCC). **p* < 0.05

**Table S19.**

|  | **Hyperactivity/Impulsivity** | | | | **Inattention** | | | | | |
| --- | --- | --- | --- | --- | --- | --- | --- | --- | --- | --- |
|  | *B* | *SE* | 95% CIs | *p* | | *B* | *SE* | 95% CIs | *p* |  |
| L DMN mPFC - L DMN PCC | 0.03 | 0.15 | -0.28:0.34 | 0.845 | | -0.04 | 0.15 | -0.34:0.26 | 0.993 |  |
| L DMN mPFC - R DMN mPFC | 0.07 | 0.15 | -0.22:0.36 | 0.845 | | -0.01 | 0.14 | -0.30:0.27 | 0.993 |  |
| L DMN mPFC - R DMN PCC | 0.08 | 0.16 | -0.23:0.39 | 0.845 | | 0 | 0.15 | -0.31:0.30 | 0.993 |  |
| L DMN PCC - R DMN mPFC | -0.17 | 0.15 | -0.48:0.13 | 0.845 | | -0.09 | 0.15 | -0.39:0.21 | 0.993 |  |
| L DMN PCC - R DMN PCC | -0.1 | 0.16 | -0.41:0.22 | 0.845 | | 0.02 | 0.15 | -0.28:0.32 | 0.993 |  |
| R DMN mPFC - R DMN PCC | -0.06 | 0.16 | -0.37:0.26 | 0.845 | | -0.04 | 0.16 | -0.35:0.28 | 0.993 |  |

**Within Default Mode Network Connectivity and Neurodevelopmental Difficulties in Comparison Children**

*Note.* Standardised beta coefficients are reported with Standard Errors (SE) and 95% Confidence Intervals (CIs), controlling for age, age^2^, gender, and in-scanner motion. Left (L), Right (R), Default Mode Network (DMN), medial Prefrontal Cortex (mPFC), and Posterior Cingulate Cortex (PCC).

**Table S20**

**Triple Network Connectivity and Neurodevelopmental Difficulties in At-Risk Children across Motion Thresholds**

|  | **Hyperactivity/Impulsivity** | | | | | | | **Inattention** | | | | | | |
| --- | --- | --- | --- | --- | --- | --- | --- | --- | --- | --- | --- | --- | --- | --- |
|  | *B* | | *SE* | | 95% CIs | *p* | | *B* | *SE* | 95% CIs | | *p* | |  |
| *0.45 threshold* |  |  | |  | |  |  | |  |  |  | |  |  |
| SN-CEN FC | 0.23 | 0.08 | | 0.08:0.38 | | 0.004** | 0.03 | | 0.08 | -0.14:0.19 | 0.752 | |  |  |
| SN-DMN FC | 0.19 | 0.08 | | 0.04:0.35 | | 0.017* | 0.06 | | 0.09 | -0.11:0.23 | 0.457 | |  |  |
| CEN-DMN FC | 0.16 | 0.08 | | -0.00:0.31 | | 0.05* | 0.07 | | 0.08 | -0.10:0.24 | 0.407 | |  |  |
| *0.4 threshold* |  |  | |  | |  |  | |  |  |  | |  |  |
| SN-CEN FC | 0.22 | 0.08 | | 0.07:0.38 | | 0.006** | 0.04 | | 0.08 | -0.13:0.20 | 0.647 | |  |  |
| SN-DMN FC | 0.19 | 0.08 | | 0.03:0.35 | | 0.021* | 0.02 | | 0.09 | -0.15:0.19 | 0.831 | |  |  |
| CEN-DMN FC | 0.17 | 0.08 | | 0.01:0.33 | | 0.033* | 0.06 | | 0.09 | -0.11:0.23 | 0.454 | |  |  |
| *0.35 threshold* |  |  | |  | |  |  | |  |  |  | |  |  |
| SN-CEN FC | 0.25 | 0.08 | | 0.08:0.41 | | 0.004** | 0.01 | | 0.09 | -0.17:0.19 | 0.938 | |  |  |
| SN-DMN FC | 0.17 | 0.09 | | -0.01:0.35 | | 0.057^+^ | 0 | | 0.09 | -0.18:0.18 | 0.981 | |  |  |
| CEN-DMN FC | 0.21 | 0.09 | | 0.04:0.38 | | 0.016* | 0.04 | | 0.09 | -0.15:0.22 | 0.695 | |  |  |
| *0.3 threshold* |  |  | |  | |  |  | |  |  |  | |  |  |
| SN-CEN FC | 0.22 | 0.09 | | 0.04:0.40 | | 0.015* | -0.02 | | 0.09 | -0.21:0.17 | 0.849 | |  |  |
| SN-DMN FC | 0.16 | 0.09 | | -0.03:0.34 | | 0.094 | 0.01 | | 0.09 | -0.18:0.19 | 0.947 | |  |  |
| CEN-DMN FC | 0.19 | 0.09 | | 0.01:0.37 | | 0.042* | -0.03 | | 0.1 | -0.22:0.16 | 0.776 | |  |  |
| *0.25 threshold* |  |  | |  | |  |  | |  |  |  | |  |  |
| SN-CEN FC | 0.21 | 0.1 | | 0.02:0.40 | | 0.035* | 0.02 | | 0.1 | -0.18:0.22 | 0.834 | |  |  |
| SN-DMN FC | 0.19 | 0.1 | | -0.01:0.39 | | 0.067^+^ | -0.03 | | 0.11 | -0.24:0.18 | 0.768 | |  |  |
| CEN-DMN FC | 0.14 | 0.1 | | -0.06:0.34 | | 0.175 | -0.05 | | 0.11 | -0.26:0.16 | 0.633 | |  |  |
| *0.2 threshold* |  |  | |  | |  |  | |  |  |  | |  |  |
| SN-CEN FC | 0.25 | 0.1 | | 0.04:0.46 | | 0.018* | 0.11 | | 0.1 | -0.10:0.31 | 0.308 | |  |  |
| SN-DMN FC | 0.24 | 0.11 | | 0.01:0.46 | | 0.043* | 0.27 | | 0.11 | 0.05:0.49 | 0.015 | |  |  |
| CEN-DMN FC | 0.09 | 0.11 | | -0.14:0.31 | | 0.441 | -0.02 | | 0.11 | -0.23:0.20 | 0.89 | |  |  |
| *0.15 threshold* |  |  | |  | |  |  | |  |  |  | |  |  |
| SN-CEN FC | 0.39 | 0.14 | | 0.11:0.67 | | 0.008** | 0.07 | | 0.15 | -0.23:0.38 | 0.631 | |  |  |
| SN-DMN FC | 0.19 | 0.17 | | -0.15:0.53 | | 0.276 | 0.41 | | 0.15 | 0.11:0.72 | 0.01* | |  |  |
| CEN-DMN FC | 0.14 | 0.16 | | -0.18:0.46 | | 0.383 | -0.1 | | 0.16 | -0.42:0.22 | 0.517 | |  |  |

*Note.* Standardised beta coefficients are reported with Standard Errors (SE) and 95% Confidence Intervals (CIs), controlling for age, age^2^, gender, and in-scanner motion. Salience Network (SN), Central Executive Network (CEN), and Default Mode Network (DMN). ^+^*p* < 0.68, **p* < 0.05, ***p* < 0.01

**Table S21**

**Triple Network Connectivity and Neurodevelopmental Difficulties in Comparison Children across Motion Thresholds**

|  | **Hyperactivity/Impulsivity** | | | | | | | **Inattention** | | | | | | |
| --- | --- | --- | --- | --- | --- | --- | --- | --- | --- | --- | --- | --- | --- | --- |
|  | *B* | | *SE* | | 95% CIs | *p* | | *B* | *SE* | 95% CIs | | *p* | |  |
| *0.45 threshold* |  |  | |  | |  |  | |  |  |  | |  |  |
| SN-CEN FC | 0.04 | 0.14 | | -0.25:0.33 | | 0.785 | -0.02 | | 0.14 | -0.30:0.26 | 0.9 | |  |  |
| SN-DMN FC | 0.07 | 0.16 | | -0.25:0.38 | | 0.669 | 0.06 | | 0.15 | -0.24:0.36 | 0.698 | |  |  |
| CEN-DMN FC | -0.54 | 0.12 | | -0.79:-0.29 | | 6.73e-05*** | -0.12 | | 0.14 | -0.40:0.17 | 0.413 | |  |  |
| *0.4 threshold* |  |  | |  | |  |  | |  |  |  | |  |  |
| SN-CEN FC | 0.04 | 0.14 | | -0.25:0.33 | | 0.785 | -0.02 | | 0.14 | -0.30:0.26 | 0.9 | |  |  |
| SN-DMN FC | 0.07 | 0.16 | | -0.25:0.38 | | 0.669 | 0.06 | | 0.15 | -0.24:0.36 | 0.698 | |  |  |
| CEN-DMN FC | -0.54 | 0.12 | | -0.79:-0.29 | | 6.73e-05*** | -0.12 | | 0.14 | -0.40:0.17 | 0.413 | |  |  |
| *0.35 threshold* |  |  | |  | |  |  | |  |  |  | |  |  |
| SN-CEN FC | -0.05 | 0.15 | | -0.36:0.25 | | 0.721 | 0.01 | | 0.15 | -0.29:0.32 | 0.942 | |  |  |
| SN-DMN FC | 0.03 | 0.16 | | -0.28:0.35 | | 0.83 | 0.1 | | 0.15 | -0.21:0.41 | 0.535 | |  |  |
| CEN-DMN FC | -0.46 | 0.12 | | -0.71:-0.22 | | 4.05e-4*** | -0.17 | | 0.14 | -0.45:0.10 | 0.21 | |  |  |
| *0.3 threshold* |  |  | |  | |  |  | |  |  |  | |  |  |
| SN-CEN FC | -0.05 | 0.15 | | -0.36:0.26 | | 0.735 | 0.02 | | 0.15 | -0.28:0.33 | 0.892 | |  |  |
| SN-DMN FC | 0.08 | 0.16 | | -0.25:0.40 | | 0.637 | 0.16 | | 0.16 | -0.16:0.48 | 0.318 | |  |  |
| CEN-DMN FC | -0.37 | 0.14 | | -0.65:-0.09 | | 0.01** | -0.13 | | 0.15 | -0.43:0.16 | 0.368 | |  |  |
| *0.25 threshold* |  |  | |  | |  |  | |  |  |  | |  |  |
| SN-CEN FC | -0.05 | 0.16 | | -0.37:0.26 | | 0.726 | 0 | | 0.16 | -0.32:0.33 | 0.991 | |  |  |
| SN-DMN FC | 0.08 | 0.17 | | -0.26:0.43 | | 0.635 | 0.12 | | 0.16 | -0.21:0.45 | 0.456 | |  |  |
| CEN-DMN FC | -0.42 | 0.14 | | -0.70:-0.13 | | 0.005** | -0.13 | | 0.15 | -0.43:0.16 | 0.365 | |  |  |
| *0.2 threshold* |  |  | |  | |  |  | |  |  |  | |  |  |
| SN-CEN FC | -0.22 | 0.17 | | -0.55:0.12 | | 0.198 | -0.03 | | 0.16 | -0.37:0.30 | 0.84 | |  |  |
| SN-DMN FC | 0.08 | 0.18 | | -0.29:0.44 | | 0.671 | 0.18 | | 0.17 | -0.16:0.53 | 0.294 | |  |  |
| CEN-DMN FC | -0.42 | 0.15 | | -0.73:-0.11 | | 0.01** | -0.23 | | 0.16 | -0.56:0.10 | 0.164 | |  |  |
| *0.15 threshold* |  |  | |  | |  |  | |  |  |  | |  |  |
| SN-CEN FC | 0.16 | 0.27 | | -0.40:0.72 | | 0.552 | -0.11 | | 0.2 | -0.54:0.31 | 0.585 | |  |  |
| SN-DMN FC | 0.14 | 0.27 | | -0.43:0.71 | | 0.61 | 0.27 | | 0.23 | -0.22:0.76 | 0.261 | |  |  |
| CEN-DMN FC | -0.02 | 0.28 | | -0.61:0.58 | | 0.95 | -0.35 | | 0.23 | -0.83:0.14 | 0.15 | |  |  |

*Note.* Standardised beta coefficients are reported with Standard Errors (SE) and 95% Confidence Intervals (CIs), controlling for age, age^2^, gender, and in-scanner motion. Salience Network (SN), Central Executive Network (CEN), and Default Mode Network (DMN). **p* < 0.05, ***p* < 0.01, ****p* < 0.001

**Table S22**

**Group Interaction on CEN-DMN Connectivity and Neurodevelopmental Difficulties across Motion Thresholds**

|  | **Hyperactivity/Impulsivity** | | | | | | |
| --- | --- | --- | --- | --- | --- | --- | --- |
| Threshold | *B* | | *SE* | | 95% CIs | *p* | |
| 0.45 | 0.65 | 0.16 | | 0.34:0.96 | | 4.31e-05*** |  |
| 0.4 | 0.66 | 0.16 | | 0.35:0.97 | | 4.34e-05*** |  |
| 0.35 | 0.64 | 0.17 | | 0.30:0.97 | | 2.09e-4*** |  |
| 0.3 | 0.5 | 0.19 | | 0.13:0.87 | | 0.008** |  |
| 0.25 | 0.63 | 0.19 | | 0.26:1.00 | | 0.001** |  |
| 0.2 | 0.64 | 0.21 | | 0.23:1.05 | | 0.002** |  |
| 0.15 | 0.06 | 0.4 | | -0.74:0.86 | | 0.882 |  |

*Note.* The threshold indicates the maximum average framewise displacement*.* Standardised beta coefficients are reported with Standard Errors (SE) and 95% Confidence Intervals (CIs), controlling for age, age^2^, gender, and in-scanner motion. ***p* < 0.01, ****p* < 0.001

**Table S23**

**Adult Network Connectivity and Neurodevelopmental Difficulties in At-Risk Children across Motion Thresholds**

|  | **Hyperactivity/Impulsivity** | | | | | | | **Inattention** | | | | | | |
| --- | --- | --- | --- | --- | --- | --- | --- | --- | --- | --- | --- | --- | --- | --- |
|  | *B* | | *SE* | | 95% CIs | *p* | | *B* | *SE* | 95% CIs | | *p* | |  |
| *0.45 threshold* |  |  | |  | |  |  | |  |  |  | |  |  |
| Ventral SN - CEN | 0.02 | 0.08 | | -0.15:0.18 | | 0.841 | 0.01 | | 0.09 | -0.16:0.19 | 0.883 | |  |  |
| Ventral SN - DMN | 0.16 | 0.08 | | 0.00:0.32 | | 0.047* | 0.03 | | 0.08 | -0.13:0.20 | 0.677 | |  |  |
| Dorsal SN - CEN | -0.07 | 0.08 | | -0.23:0.09 | | 0.397 | 0.13 | | 0.08 | -0.04:0.30 | 0.125 | |  |  |
| Dorsal SN - DMN | 0.13 | 0.08 | | -0.03:0.29 | | 0.111 | 0 | | 0.08 | -0.16:0.16 | 0.992 | |  |  |
| CEN - DMN | 0.2 | 0.08 | | 0.05:0.36 | | 0.012* | 0.12 | | 0.08 | -0.04:0.28 | 0.149 | |  |  |
| *0.4 threshold* |  |  | |  | |  |  | |  |  |  | |  |  |
| Ventral SN - CEN | 0 | 0.08 | | -0.16:0.17 | | 0.981 | 0.01 | | 0.09 | -0.17:0.18 | 0.954 | |  |  |
| Ventral SN - DMN | 0.15 | 0.08 | | -0.01:0.32 | | 0.068^+^ | 0.03 | | 0.09 | -0.14:0.20 | 0.755 | |  |  |
| Dorsal SN - CEN | -0.07 | 0.08 | | -0.24:0.10 | | 0.402 | 0.1 | | 0.09 | -0.07:0.27 | 0.243 | |  |  |
| Dorsal SN - DMN | 0.14 | 0.08 | | -0.03:0.30 | | 0.103 | 0 | | 0.09 | -0.17:0.17 | 1 | |  |  |
| CEN - DMN | 0.23 | 0.08 | | 0.08:0.39 | | 0.004** | 0.15 | | 0.08 | -0.02:0.31 | 0.084 | |  |  |
| *0.35 threshold* |  |  | |  | |  |  | |  |  |  | |  |  |
| Ventral SN - CEN | -0.01 | 0.09 | | -0.19:0.17 | | 0.927 | -0.02 | | 0.1 | -0.21:0.17 | 0.833 | |  |  |
| Ventral SN - DMN | 0.12 | 0.09 | | -0.05:0.30 | | 0.165 | 0.01 | | 0.09 | -0.17:0.19 | 0.916 | |  |  |
| Dorsal SN - CEN | -0.08 | 0.09 | | -0.26:0.09 | | 0.348 | 0.09 | | 0.09 | -0.10:0.27 | 0.344 | |  |  |
| Dorsal SN - DMN | 0.07 | 0.09 | | -0.10:0.25 | | 0.4 | -0.04 | | 0.09 | -0.22:0.15 | 0.694 | |  |  |
| CEN - DMN | 0.27 | 0.08 | | 0.10:0.43 | | 0.002** | 0.12 | | 0.09 | -0.06:0.29 | 0.192 | |  |  |
| *0.3 threshold* |  |  | |  | |  |  | |  |  |  | |  |  |
| Ventral SN - CEN | -0.02 | 0.09 | | -0.20:0.17 | | 0.868 | -0.03 | | 0.1 | -0.22:0.17 | 0.797 | |  |  |
| Ventral SN - DMN | 0.11 | 0.09 | | -0.07:0.29 | | 0.247 | -0.05 | | 0.09 | -0.23:0.14 | 0.618 | |  |  |
| Dorsal SN - CEN | -0.08 | 0.09 | | -0.26:0.10 | | 0.398 | 0.1 | | 0.1 | -0.10:0.29 | 0.32 | |  |  |
| Dorsal SN - DMN | 0.08 | 0.09 | | -0.10:0.27 | | 0.387 | -0.04 | | 0.1 | -0.23:0.15 | 0.705 | |  |  |
| CEN - DMN | 0.23 | 0.09 | | 0.06:0.41 | | 0.009** | 0.13 | | 0.09 | -0.06:0.31 | 0.18 | |  |  |
| *0.25 threshold* |  |  | |  | |  |  | |  |  |  | |  |  |
| Ventral SN - CEN | 0.05 | 0.1 | | -0.16:0.25 | | 0.662 | -0.01 | | 0.11 | -0.23:0.21 | 0.927 | |  |  |
| Ventral SN - DMN | -0.02 | 0.1 | | -0.23:0.18 | | 0.813 | -0.04 | | 0.11 | -0.25:0.18 | 0.736 | |  |  |
| Dorsal SN - CEN | -0.09 | 0.1 | | -0.29:0.10 | | 0.35 | 0.08 | | 0.11 | -0.13:0.30 | 0.451 | |  |  |
| Dorsal SN - DMN | 0 | 0.1 | | -0.20:0.21 | | 0.976 | -0.04 | | 0.11 | -0.26:0.17 | 0.689 | |  |  |
| CEN - DMN | 0.14 | 0.1 | | -0.06:0.33 | | 0.164 | 0.11 | | 0.1 | -0.09:0.31 | 0.293 | |  |  |
| *0.2 threshold* |  |  | |  | |  |  | |  |  |  | |  |  |
| Ventral SN - CEN | 0.09 | 0.11 | | -0.13:0.32 | | 0.405 | 0 | | 0.11 | -0.22:0.22 | 0.989 | |  |  |
| Ventral SN - DMN | -0.02 | 0.11 | | -0.25:0.20 | | 0.841 | 0.14 | | 0.11 | -0.08:0.36 | 0.209 | |  |  |
| Dorsal SN - CEN | -0.01 | 0.11 | | -0.24:0.21 | | 0.905 | -0.04 | | 0.11 | -0.25:0.17 | 0.712 | |  |  |
| Dorsal SN - DMN | -0.03 | 0.11 | | -0.25:0.19 | | 0.807 | -0.01 | | 0.11 | -0.22:0.20 | 0.928 | |  |  |
| CEN - DMN | 0.1 | 0.11 | | -0.11:0.32 | | 0.342 | 0.15 | | 0.1 | -0.06:0.36 | 0.151 | |  |  |
| *0.15 threshold* |  |  | |  | |  |  | |  |  |  | |  |  |
| Ventral SN - CEN | 0.23 | 0.15 | | -0.07:0.54 | | 0.131 | -0.04 | | 0.15 | -0.35:0.27 | 0.791 | |  |  |
| Ventral SN - DMN | -0.03 | 0.17 | | -0.37:0.31 | | 0.869 | 0.31 | | 0.16 | -0.01:0.63 | 0.058 | |  |  |
| Dorsal SN - CEN | -0.03 | 0.16 | | -0.36:0.29 | | 0.841 | -0.15 | | 0.16 | -0.46:0.17 | 0.349 | |  |  |
| Dorsal SN - DMN | -0.14 | 0.17 | | -0.49:0.21 | | 0.428 | 0.19 | | 0.16 | -0.14:0.52 | 0.244 | |  |  |
| CEN - DMN | 0.1 | 0.16 | | -0.22:0.42 | | 0.541 | 0.1 | | 0.16 | -0.23:0.42 | 0.556 | |  |  |

*Note.* Standardised beta coefficients are reported with Standard Errors (SE) and 95% Confidence Intervals (CIs), controlling for age, age^2^, gender, and in-scanner motion. Salience Network (SN), Central Executive Network (CEN), and Default Mode Network (DMN). ^+^*p* < 0.069, **p* < 0.05, ***p* < 0.01

**Table S24**

**Adult Network Connectivity and Neurodevelopmental Difficulties Comparison Children across Motion Thresholds**

|  | **Hyperactivity/Impulsivity** | | | | | | | **Inattention** | | | |
| --- | --- | --- | --- | --- | --- | --- | --- | --- | --- | --- | --- |
|  | *B* | | *SE* | | 95% CIs | *p* | | *B* | *SE* | 95% CIs | *p* |
| *0.45 threshold* |  |  | |  | |  |  | |  |  |  |
| Ventral SN - CEN | 0.15 | 0.15 | | -0.15:0.46 | | 0.317 | 0 | | 0.16 | -0.31:0.31 | 0.998 |
| Ventral SN - DMN | -0.16 | 0.14 | | -0.45:0.13 | | 0.281 | -0.13 | | 0.14 | -0.41:0.16 | 0.385 |
| Dorsal SN - CEN | 0.14 | 0.15 | | -0.15:0.44 | | 0.334 | 0.07 | | 0.14 | -0.22:0.36 | 0.63 |
| Dorsal SN - DMN | -0.09 | 0.14 | | -0.39:0.20 | | 0.515 | -0.09 | | 0.14 | -0.38:0.19 | 0.506 |
| CEN - DMN | -0.22 | 0.14 | | -0.50:0.05 | | 0.11 | -0.18 | | 0.14 | -0.45:0.10 | 0.196 |
| *0.4 threshold* |  |  | |  | |  |  | |  |  |  |
| Ventral SN - CEN | 0.15 | 0.15 | | -0.15:0.46 | | 0.317 | 0 | | 0.16 | -0.31:0.31 | 0.998 |
| Ventral SN - DMN | -0.16 | 0.14 | | -0.45:0.13 | | 0.281 | -0.13 | | 0.14 | -0.41:0.16 | 0.385 |
| Dorsal SN - CEN | 0.14 | 0.15 | | -0.15:0.44 | | 0.334 | 0.07 | | 0.14 | -0.22:0.36 | 0.63 |
| Dorsal SN - DMN | -0.09 | 0.14 | | -0.39:0.20 | | 0.515 | -0.09 | | 0.14 | -0.38:0.19 | 0.506 |
| CEN - DMN | -0.22 | 0.14 | | -0.50:0.05 | | 0.11 | -0.18 | | 0.14 | -0.45:0.10 | 0.196 |
| *0.35 threshold* |  |  | |  | |  |  | |  |  |  |
| Ventral SN - CEN | 0.11 | 0.15 | | -0.20:0.41 | | 0.475 | 0.05 | | 0.16 | -0.27:0.37 | 0.752 |
| Ventral SN - DMN | -0.15 | 0.15 | | -0.44:0.14 | | 0.307 | -0.14 | | 0.14 | -0.43:0.14 | 0.317 |
| Dorsal SN - CEN | 0.06 | 0.15 | | -0.25:0.36 | | 0.706 | 0.11 | | 0.15 | -0.19:0.40 | 0.464 |
| Dorsal SN - DMN | -0.03 | 0.14 | | -0.32:0.26 | | 0.826 | -0.12 | | 0.14 | -0.40:0.16 | 0.4 |
| CEN - DMN | -0.17 | 0.13 | | -0.44:0.10 | | 0.213 | -0.2 | | 0.13 | -0.47:0.07 | 0.144 |
| *0.3 threshold* |  |  | |  | |  |  | |  |  |  |
| Ventral SN - CEN | 0.07 | 0.15 | | -0.24:0.38 | | 0.661 | 0.03 | | 0.16 | -0.28:0.35 | 0.83 |
| Ventral SN - DMN | -0.15 | 0.15 | | -0.46:0.15 | | 0.311 | -0.14 | | 0.15 | -0.43:0.16 | 0.346 |
| Dorsal SN - CEN | 0.06 | 0.15 | | -0.23:0.35 | | 0.678 | 0.11 | | 0.14 | -0.18:0.40 | 0.454 |
| Dorsal SN - DMN | -0.02 | 0.15 | | -0.32:0.28 | | 0.903 | -0.1 | | 0.14 | -0.39:0.18 | 0.471 |
| CEN - DMN | -0.16 | 0.14 | | -0.45:0.13 | | 0.27 | -0.2 | | 0.14 | -0.48:0.09 | 0.17 |
| *0.25 threshold* |  |  | |  | |  |  | |  |  |  |
| Ventral SN - CEN | -0.02 | 0.16 | | -0.35:0.31 | | 0.907 | -0.02 | | 0.17 | -0.35:0.32 | 0.919 |
| Ventral SN - DMN | -0.11 | 0.16 | | -0.44:0.22 | | 0.496 | -0.14 | | 0.15 | -0.45:0.17 | 0.375 |
| Dorsal SN - CEN | 0.01 | 0.15 | | -0.30:0.32 | | 0.951 | 0.09 | | 0.15 | -0.22:0.39 | 0.574 |
| Dorsal SN - DMN | -0.02 | 0.16 | | -0.34:0.31 | | 0.916 | -0.12 | | 0.15 | -0.43:0.18 | 0.418 |
| CEN - DMN | -0.2 | 0.16 | | -0.52:0.12 | | 0.205 | -0.23 | | 0.15 | -0.53:0.08 | 0.143 |
| *0.2 threshold* |  |  | |  | |  |  | |  |  |  |
| Ventral SN - CEN | 0.08 | 0.17 | | -0.27:0.43 | | 0.643 | -0.02 | | 0.18 | -0.38:0.33 | 0.889 |
| Ventral SN - DMN | -0.11 | 0.18 | | -0.47:0.24 | | 0.525 | -0.14 | | 0.16 | -0.48:0.19 | 0.39 |
| Dorsal SN - CEN | 0.07 | 0.17 | | -0.28:0.42 | | 0.706 | 0.03 | | 0.17 | -0.32:0.37 | 0.871 |
| Dorsal SN - DMN | -0.07 | 0.17 | | -0.41:0.28 | | 0.705 | -0.12 | | 0.16 | -0.45:0.21 | 0.457 |
| CEN - DMN | -0.24 | 0.17 | | -0.58:0.10 | | 0.164 | -0.33 | | 0.17 | -0.66:0.01 | 0.058^+^ |
| *0.15 threshold* |  |  | |  | |  |  | |  |  |  |
| Ventral SN - CEN | 0 | 0.28 | | -0.59:0.59 | | 0.992 | -0.15 | | 0.24 | -0.65:0.35 | 0.54 |
| Ventral SN - DMN | 0.16 | 0.25 | | -0.35:0.68 | | 0.514 | -0.1 | | 0.21 | -0.54:0.35 | 0.648 |
| Dorsal SN - CEN | 0.08 | 0.29 | | -0.54:0.69 | | 0.799 | -0.33 | | 0.21 | -0.76:0.11 | 0.133 |
| Dorsal SN - DMN | 0.17 | 0.26 | | -0.37:0.70 | | 0.522 | -0.01 | | 0.21 | -0.45:0.42 | 0.953 |
| CEN - DMN | 0.12 | 0.28 | | -0.47:0.71 | | 0.664 | -0.48 | | 0.18 | -0.87:-0.10 | 0.016* |

*Note.* Standardised beta coefficients are reported with Standard Errors (SE) and 95% Confidence Intervals (CIs), controlling for age, age^2^, gender, and in-scanner motion. Salience Network (SN), Central Executive Network (CEN), and Default Mode Network (DMN). ^+^*p* < 0.059, **p* < 0.05

**Table S25**

**Group Interaction on Adult CEN-DMN Connectivity and Neurodevelopmental Difficulties across Motion Thresholds**

|  | **Hyperactivity/Impulsivity** | | | | | | |
| --- | --- | --- | --- | --- | --- | --- | --- |
| Threshold | *B* | | *SE* | | 95% CIs | *p* | |
| 0.45 | 0.37 | 0.16 | | 0.06:0.68 | | 0.021* |  |
| 0.4 | 0.39 | 0.16 | | 0.07:0.70 | | 0.016* |  |
| 0.35 | 0.37 | 0.17 | | 0.03:0.70 | | 0.032* |  |
| 0.3 | 0.35 | 0.18 | | -0.00:0.69 | | 0.051^+^ |  |
| 0.25 | 0.4 | 0.19 | | 0.03:0.77 | | 0.036* |  |
| 0.2 | 0.42 | 0.21 | | 0.01:0.83 | | 0.046* |  |
| 0.15 | -0.31 | 0.4 | | -1.11:0.49 | | 0.444 |  |

*Note.* The threshold indicates the maximum average framewise displacement*.* Standardised beta coefficients are reported with Standard Errors (SE) and 95% Confidence Intervals (CIs), controlling for age, age^2^, gender, and in-scanner motion. ^+^*p* < 0.052, **p* < 0.05

**Table S26**

**Network Connectivity Differences between the Samples over Motion Thresholds**

|  | At-Risk | Comparison | t-test | | | Regression | | | |
| --- | --- | --- | --- | --- | --- | --- | --- | --- | --- |
|  | *M* (*SD*) | *M* (*SD*) | *t* | *D* | *p* | *β* | *SE* | CI | *p* |
| *0.45 threshold* |  |  |  |  |  |  |  |  |  |
| SN - CEN | 0.11 (0.17) | 0.06 (0.16) | -2.13 | -0.33 | 0.034* | -0.2 | 0.15 | -0.51:0.10 | 0.194 |
| SN - DMN | 0.17 (0.20) | 0.19 (0.21) | 0.74 | 0.11 | 0.46 | 0.08 | 0.16 | -0.23:0.40 | 0.598 |
| CEN - DMN | 0.19 (0.15) | 0.17 (0.16) | -0.82 | -0.12 | 0.411 | -0.03 | 0.16 | -0.34:0.28 | 0.858 |
| *0.4 threshold* |  |  |  |  |  |  |  |  |  |
| SN - CEN | 0.11 (0.17) | 0.06 (0.16) | -1.94 | -0.3 | 0.054^+^ | -0.19 | 0.15 | -0.49:0.12 | 0.229 |
| SN - DMN | 0.16 (0.19) | 0.19 (0.21) | 0.84 | 0.13 | 0.404 | 0.09 | 0.16 | -0.23:0.40 | 0.595 |
| CEN - DMN | 0.19 (0.15) | 0.17 (0.16) | -0.72 | -0.11 | 0.473 | -0.03 | 0.16 | -0.34:0.28 | 0.835 |
| *0.35 threshold* |  |  |  |  |  |  |  |  |  |
| SN - CEN | 0.09 (0.16) | 0.05 (0.15) | -1.77 | -0.28 | 0.079 | -0.23 | 0.16 | -0.55:0.10 | 0.17 |
| SN - DMN | 0.15 (0.18) | 0.18 (0.21) | 0.99 | 0.15 | 0.325 | 0.06 | 0.17 | -0.27:0.40 | 0.702 |
| CEN - DMN | 0.18 (0.15) | 0.17 (0.17) | -0.08 | -0.01 | 0.94 | 0.05 | 0.17 | -0.27:0.38 | 0.755 |
| *0.3 threshold* |  |  |  |  |  |  |  |  |  |
| SN - CEN | 0.10 (0.15) | 0.05 (0.16) | -2.02 | -0.32 | 0.045* | -0.29 | 0.17 | -0.62:0.04 | 0.086 |
| SN - DMN | 0.14 (0.18) | 0.20 (0.20) | 1.77 | 0.28 | 0.079 | 0.17 | 0.17 | -0.16:0.51 | 0.311 |
| CEN - DMN | 0.17 (0.14) | 0.16 (0.15) | -0.38 | -0.06 | 0.704 | -0.03 | 0.17 | -0.37:0.31 | 0.872 |
| *0.25 threshold* |  |  |  |  |  |  |  |  |  |
| SN - CEN | 0.10 (0.15) | 0.02 (0.13) | -3.31 | -0.59 | 0.001*** | -0.55 | 0.18 | -0.90:-0.20 | 0.002** |
| SN - DMN | 0.15 (0.18) | 0.19 (0.22) | 1.22 | 0.2 | 0.223 | 0.11 | 0.18 | -0.24:0.47 | 0.529 |
| CEN - DMN | 0.18 (0.14) | 0.15 (0.16) | -0.99 | -0.16 | 0.324 | -0.1 | 0.18 | -0.46:0.26 | 0.58 |
| *0.2 threshold* |  |  |  |  |  |  |  |  |  |
| SN - CEN | 0.11 (0.15) | 0.02 (0.16) | -3.15 | -0.57 | 0.002** | -0.49 | 0.18 | -0.85:-0.13 | 0.008** |
| SN - DMN | 0.17 (0.16) | 0.19 (0.22) | 0.67 | 0.12 | 0.503 | 0.04 | 0.19 | -0.34:0.42 | 0.823 |
| CEN - DMN | 0.17 (0.13) | 0.15 (0.15) | -0.96 | -0.17 | 0.338 | -0.1 | 0.19 | -0.48:0.28 | 0.602 |
| *0.15 threshold* |  |  |  |  |  |  |  |  |  |
| SN - CEN | 0.09 (0.15) | 0.01 (0.13) | -2.58 | -0.62 | 0.012* | -0.59 | 0.23 | -1.05:-0.13 | 0.013* |
| SN - DMN | 0.18 (0.17) | 0.22 (0.20) | 0.89 | 0.21 | 0.374 | 0.12 | 0.24 | -0.37:0.61 | 0.62 |
| CEN - DMN | 0.17 (0.13) | 0.10 (0.12) | -2.05 | -0.51 | 0.044 | -0.43 | 0.25 | -0.92:0.06 | 0.086 |

*Note.* The threshold indicates the maximum average framewise displacement*.* Standardised beta coefficients are reported with Standard Errors (SE) and 95% Confidence Intervals (CIs), controlling for age, age^2^, gender, and in-scanner motion. Cohen’s D (*D*), Salience Network (SN), Central Executive Network (CEN), Default Mode Network (DMN). ^+^*p* < 0.055, **p* < 0.05, ***p* < 0.01, ****p* < 0.01

**Table S27**

**Network Connectivity Differences in ADHD over Motion Thresholds**

|  | ADHD | Comparison | t-test | | | Regression | | | |
| --- | --- | --- | --- | --- | --- | --- | --- | --- | --- |
|  | *M* (*SD*) | *M* (*SD*) | *t* | *D* | *p* | *β* | *SE* | CI | *p* |
| *0.45 threshold* |  |  |  |  |  |  |  |  |  |
| SN - CEN | 0.16 (0.13) | 0.06 (0.16) | -3.08 | -0.7 | 0.003** | -0.56 | 0.24 | -1.04:-0.08 | 0.023* |
| SN - DMN | 0.23 (0.17) | 0.19 (0.21) | -0.78 | -0.18 | 0.44 | -0.15 | 0.26 | -0.67:0.36 | 0.554 |
| CEN - DMN | 0.24 (0.08) | 0.18 (0.16) | -1.73 | -0.45 | 0.087 | -0.18 | 0.27 | -0.71:0.36 | 0.512 |
| *0.4 threshold* |  |  |  |  |  |  |  |  |  |
| SN - CEN | 0.16 (0.13) | 0.06 (0.16) | -3.08 | -0.7 | 0.003** | -0.56 | 0.24 | -1.04:-0.08 | 0.023* |
| SN - DMN | 0.23 (0.17) | 0.19 (0.21) | -0.78 | -0.18 | 0.44 | -0.15 | 0.26 | -0.67:0.36 | 0.554 |
| CEN - DMN | 0.24 (0.08) | 0.18 (0.16) | -1.73 | -0.45 | 0.087 | -0.18 | 0.27 | -0.71:0.36 | 0.512 |
| *0.35 threshold* |  |  |  |  |  |  |  |  |  |
| SN - CEN | 0.17 (0.11) | 0.05 (0.15) | -3.47 | -0.88 | 0.001*** | -0.75 | 0.25 | -1.26:-0.25 | 0.004** |
| SN - DMN | 0.22 (0.17) | 0.19 (0.21) | -0.72 | -0.17 | 0.476 | -0.24 | 0.27 | -0.78:0.31 | 0.39 |
| CEN - DMN | 0.23 (0.08) | 0.17 (0.17) | -1.51 | -0.41 | 0.135 | -0.13 | 0.27 | -0.68:0.41 | 0.629 |
| *0.3 threshold* |  |  |  |  |  |  |  |  |  |
| SN - CEN | 0.15 (0.13) | 0.05 (0.16) | -2.77 | -0.69 | 0.007** | -0.64 | 0.26 | -1.16:-0.12 | 0.017* |
| SN - DMN | 0.21 (0.17) | 0.21 (0.19) | 0.03 | 0.01 | 0.98 | -0.15 | 0.28 | -0.70:0.40 | 0.592 |
| CEN - DMN | 0.22 (0.08) | 0.16 (0.15) | -1.75 | -0.5 | 0.085 | -0.21 | 0.29 | -0.78:0.37 | 0.472 |
| *0.25 threshold* |  |  |  |  |  |  |  |  |  |
| SN - CEN | 0.20 (0.11) | 0.02 (0.13) | -5.75 | -1.55 | 2.52E-07*** | -1.31 | 0.25 | -1.80:-0.82 | 1.52E-06*** |
| SN - DMN | 0.21 (0.17) | 0.21 (0.20) | -0.17 | -0.04 | 0.863 | -0.16 | 0.29 | -0.74:0.43 | 0.597 |
| CEN - DMN | 0.19 (0.10) | 0.15 (0.16) | -1.05 | -0.3 | 0.299 | -0.05 | 0.3 | -0.66:0.55 | 0.857 |
| *0.2 threshold* |  |  |  |  |  |  |  |  |  |
| SN - CEN | 0.20 (0.10) | 0.03 (0.16) | -4.24 | -1.3 | 7.47E-05*** | -1.13 | 0.28 | -1.70:-0.56 | 2.00E-4*** |
| SN - DMN | 0.21 (0.18) | 0.20 (0.22) | -0.32 | -0.09 | 0.747 | -0.22 | 0.32 | -0.86:0.41 | 0.489 |
| CEN - DMN | 0.21 (0.08) | 0.14 (0.16) | -1.72 | -0.55 | 0.091 | -0.32 | 0.33 | -0.97:0.33 | 0.334 |
| *0.15 threshold* |  |  |  |  |  |  |  |  |  |
| SN - CEN | 0.24 (0.06) | 0.01 (0.15) | -3.87 | -1.96 | 4.86E-04*** | -1.52 | 0.43 | -2.40:-0.63 | 0.002** |
| SN - DMN | 0.20 (0.19) | 0.25 (0.18) | 0.74 | 0.28 | 0.466 | 0.25 | 0.48 | -0.74:1.23 | 0.61 |
| CEN - DMN | 0.24 (0.09) | 0.09 (0.13) | -2.94 | -1.25 | 0.006** | -0.91 | 0.44 | -1.82:-0.00 | 0.049* |

*Note.* The threshold indicates the maximum average framewise displacement*.* Standardised beta coefficients are reported with Standard Errors (SE) and 95% Confidence Intervals (CIs), controlling for age, age^2^, gender, and in-scanner motion. Cohen’s D (*D*), Salience Network (SN), Central Executive Network (CEN), Default Mode Network (DMN). **p* < 0.05, ***p* < 0.01, ****p* < 0.01
